# Bromocriptine Mesylate Attenuates Amyotrophic Lateral Sclerosis: A Phase 2a, Randomized, Double-Blind, Placebo-Controlled Research in Japanese Patients

**DOI:** 10.1371/journal.pone.0149509

**Published:** 2016-02-24

**Authors:** Eiichiro Nagata, Mieko Ogino, Kounosuke Iwamoto, Yasuhisa Kitagawa, Yasuo Iwasaki, Fumihito Yoshii, Joh-E. Ikeda, ALS Consortium Investigators

**Affiliations:** 1 Department of Neurology, Tokai University School of Medicine, Isehara, Japan; 2 Department of Neurology, Kitasato University School of Medicine, Sagamihara, Japan; 3 Department of Neurology, Toho University Omori Medical Center, Tokyo, Japan; 4 Department of Neurology, Tokai University Hachioji Hospital, Tokyo, Japan; 5 Department of Neurology, Tokai University Oiso Hospital, Kanagawa, Japan; 6 Molecular Neurology, Faculty of Medicine, Kitasato University School of Medicine, Sagamihara, Japan; 7 Department of Pediatrics, Faculty of Medicine, University of Ottawa, ARC/Children’s Hospital of Eastern Ontario, Ottawa, Canada; National Health Research Institutes, TAIWAN

## Abstract

**Objective:**

Bromocriptine mesylate (BRC), a dopamine D2 receptor agonist has been shown to confer neuroprotection, sustained motor function and slowed disease progression in mouse models of amyotrophic lateral sclerosis (ALS) Here we report a first in human trial in ALS.

**Design:**

A multicenter, Riluzole add-on, randomized, double-blind, placebo controlled 102-week extension BRC clinical trial.

**Methods:**

The trial was conducted between January 2009 and March 2012 on 36 Japanese ALS patients. A 12-week treatment with Riluzole observational period was followed by combined treatment (Riluzole + BRC; n = 29 or Riluzole + placebo; n = 7). The dosing commenced at 1.25 mg/day increasing in steps at two weeks intervals to a maximum of 15 mg/day. The efficacy of BRC was evaluated by comparing BRC and placebo groups upon completion of stepwise dosing at 14 weeks 2 points (1^st^ endpoint) and upon completion or discontinuation of the study (2^nd^ endpoint) of the dosing.

**Results:**

Statistics analyses revealed a marginal BRC treatment efficacy with P≦20%to placebo by 1^st^ and 2^nd^ endpoint analysis. In the 1^st^ endpoint analysis, BRC group was significantly effective on the scores of ALSAQ40-communicaton (P = 1.2%), eating and drinking (P = 2.2%), ALSFRS-R total (P = 17.6%), grip strength (P = 19.8%) compared to the placebo group. In the 2^nd^ endpoint analysis, differences between the scores of Limb Norris Scale (P = 18.3%), ALSAQ40-communication (P = 11.9%), eating and drinking (P = 13.6%), and neck forward-bent test (P = 15.4%) of BRC group were detected between the two groups. There was no significant difference between the treatment groups for adverse events or serious drug reactions incidence.

**Conclusions:**

BRC sustains motoneuronal function at least in part through BRC treatment. Further analysis involving a Phase 2b or 3 clinical trial is required but BRC currently shows promise for ALS treatment.

**Trial Registration:**

UMIN Clinical Trials UMIN000008527

## Introduction

Amyotrophic lateral sclerosis (ALS) is a neurodegenerative disorder that preferentially targets motor neurons controlling muscle movement and is usually fatal. Approximatley 90% of ALS cases occur sporadically in the absence of a clear family history, the remainder have a clear genetic background and are diagnosed as familial ALS [1,2]. While the precise etiology or etiologies of ALS remains unknown, a complex interplay of many pathogenic factors, including oxidative stress, excitotoxicity, mitochondrial dysfunction, disruption of neurofilament network, neural inflammation, non-cell autonomous damage and protein aggregations such as mutant SOD1, TDP-43, and FUS proteins, have been suggested as possible potential factors [3–6]. Notably, an elevation of reactive oxygen species (ROS) possibly reflecting mitochondrial dysfunction [3,6], the reduction of glutamate uptake in motor neurons, and induction of oxidation in neighboring astrocytes [7] point to roles for oxidative stress and inflammatory response in ALS [8]. We believe that oxidative stress therefore represents a credible target for the development of novel therapeutic agents for ALS. We have previously developed the neuronal apoptosis inhibitory protein (NAIP)-ELISA-based drug screening system [9]. NAIP is a founding member of anti-apoptosis IAP family [10] and selectively suppresses oxidative stress-induced cell death. Both upregulation and exogenous over expression of NAIP protects neuronal cells against oxidative stress in vivo and in vitro [11–13]. Using this system, we have identified several compounds that transiently upregulate NAIP, including bromocriptine methylate (BRC) [9,13]. BRC confered protection against oxidative stress-induced cell death [14,15]. Currently the only Food and Drug Administration (FDA)-approved drug for the treatment of ALS, the anti-glutamatergic Riluzole, confers only a modest therapeutic efficacy [16,17]. Therefore, there is a clear unmet need for effective ALS therapeutic interventions [18]. The present study is designed to evaluate both the efficacy of BRC in the presence of Riluzole on motor function and quality of life (QOL) as well as safety in Japanese patients with solitary ALS.

## Materials and Methods

### Participants and study design

A double blind, Riluzole add-on placebo controlled BRC safety and efficacy trial in Japanese ALS patients was designed. The study protocol (08R-103) was approved by the Institutional Review Board for Clinical Research, Tokai University, followed the tenets of the Declaration of Helsinki and is registered at UMIN (UMIN000008527). The approved protocol for this trial and supporting CONSORT checklist are available as supporting information; see S1 Protocol and S1 CONSORT Checklist. All participants provided written informed consent. This is a Phase 2a clinical trial to evaluate the safety and the efficacy of BRC. We determined 50 ALS patients who lived around Tokyo metropolitan area suburbs in Japan.

**Fig 1:**
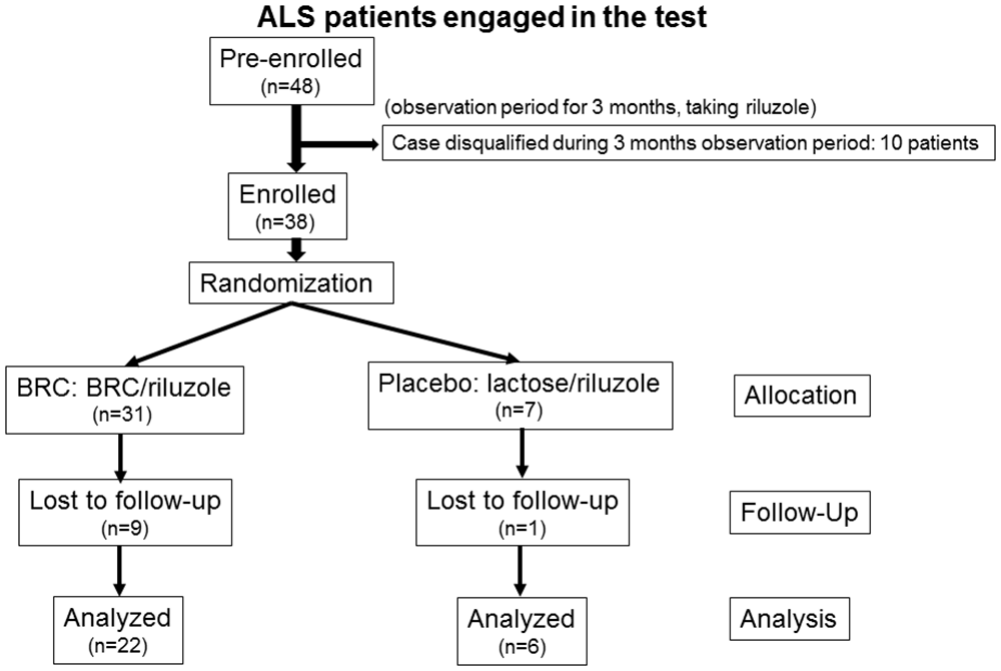
Trial profile. BRC: bromocriptine methylate.

48 ALS patients were recruited from 3 university hospitals and 4 general hospitals in Japan between January 1^st^, 2009 and March 31^th^, 2012. We enrolled patients with a diagnosis of possible, laboratory-supported probable, probable, or definite ALS in accordance with the revised El Escorial Modified Airlie House Diagnostic criteria [19]. ALS was diagnosed in all study entrants within 3 years of the inception of our study. The forced vital capacity (FVC) of patients was more than 70%. The ages of patients were between 20 and 75 at the time of taking informed consent. The changes in ALSFRS-R scores of patients were between -1 and -4 during the pretreatment 12-week observation period. All study entrants were treated with 100mg of Riluszole (50mg a twice daily) during the course of this study. 10 participants were disqualified during 12 weeks observation period (Fig 1). The final number of participants was 38 (BRC group: n = 31, control (placebo group: n = 7)). 10 participants were withdrawn during the course of study. Eight participants withdrew from the BRC group following adverse events such as nausea, constipation, there was one death in the placebo group, and one participant in the BRC group left the study of his own accord. In the placebo group, one participant died secondary to ALS progression. In the full analysis set (FAS) to efficacy assessment, 2 patients were disqualified (BRC n = 2, placebo n = 0). In one patient, his secrecy obligation was not defended. In other patient, we could not get the validity at all about this medicine. In per protocol set (PPS) to efficacy assessment, there were no unqualified patients. No significant differences between BRC and placebo groups was detected for the baseline characteristics including age, gender, body weight, BMI, ALS duration, family history, ALSFRS-R score, and vital capacity (Table 1).

**Table 1:** Summary of demographic and baseline characteristics for randomized groups in BRC and placebo.

	BRC (n = 29)	Placebo (n = 7)
Age/ years	59.7(±9.0)	58.7(±10.1)
Male	21(72.4%)	6(85.7%)
Female	8(27.6%)	1(14.3%)
Body weight/ Kg	58.08(±9.47)	58.97(±7.53)
Body-mass index/ kg/m2	21.9	21.6
Duration of symptoms/ years	1.45(±0.69)	1.60(±0.77)
Family history of amyotrophic lateral sclerosis	NA	NA
Baseline ALSFRS-R score	40.0(±5.0)	39.9(±5.6)
Mean vital capacity at baseline	105.03(±20.30)	92.19(±14.89)
ALS severity/ patients		
I	8(27.6%)	1(14.3%)
II	11(37.9%)	3(42.9%)
III	10(34.5%)	3(42.9%)
unknown	0(0.0%)	0(0.0%)

We classified clinical manners of ALS severity of five grades established by Japanese Ministry of Health, labor and Welfare as follows.

ALS severity

I: Housekeeping and working are possible in general.

II: Although housekeeping and working are impossible, daily life becomes independent in general.

III: Meal, excretion, and any one or more movements, but daily life takes care.

IV: Breathing difficulty, sputum excretion difficulty, or swallowing difficulty.

### Randomisation and masking

This investigator initiated study was designed as a Riluzole add-on, randomized, double-blind, placebo-controlled trial. Efficacy and safety were evaluated through analysis of change in each variable measured at the beginning of the observation period and at the commencement and the end of the treatment with BRC/ Riluzole/ lactose (BRC arm) and Riluzole/ lactose (placebo arm). The participants were blinded and we randomly allocated them in a 4: 1 ratio to receive each drugs using a computer-generated random allocation sequence.

### Procedures

**Fig 2:**
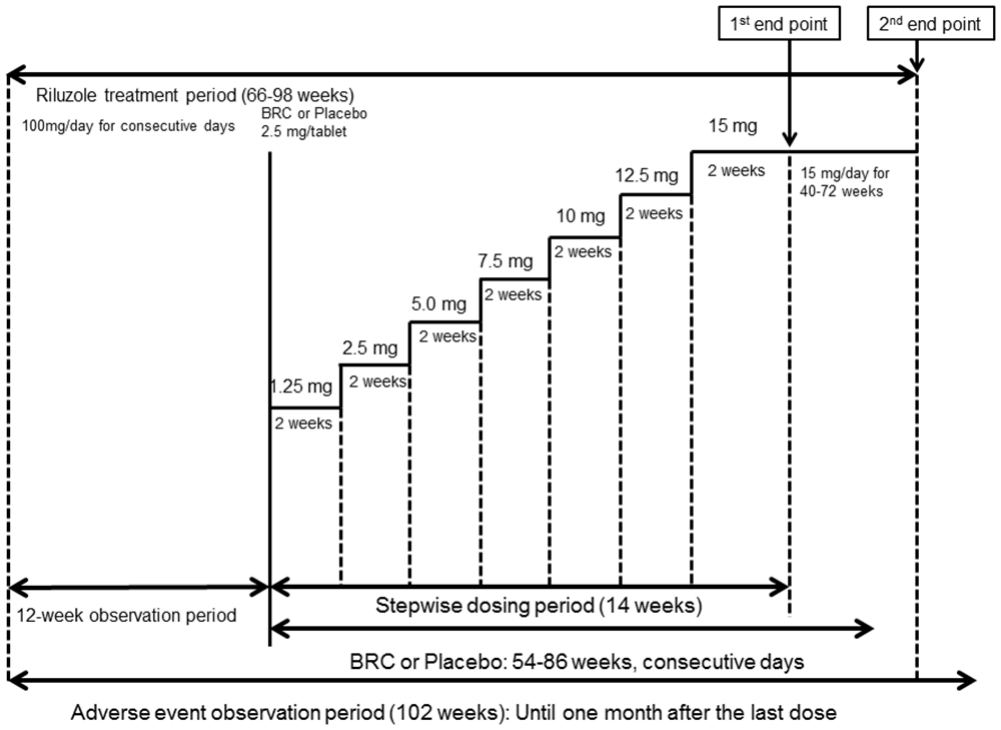
Flow chart of the clinical study (drug treatment method): BRC stepwise dosing schedule. There was no significant difference on each heading between BRC and Placebo groups.

For patients who had been dosed Riluzole 4 weeks prior or on time of the observation period, the 12-week treatment with 100mg Riluzole alone (50mg twice daily) during the observation period was performed and followed by the either BRC or placebo treatments. The initial dose of 1.25 mg BRC per day was increased to 15 mg/day at 2-week intervals over 12-weeks (Fig 2). The dosing period from 58 to 90 week ended in a 4 week stepwise dose reduction followed by a one month observational period following the completion of dosing. When a dose-related adverse effect was observed rendering the, for example, 10mg/day difficult to maintain, a reduced dose of 7.5 mg/day was used as the maintenance dose. One patient took 10mg/day of BRC as the maintenance dose for his nausea.

Statistical analyses of ALSFRS-R, %FVC, modified Norris scale (limb and bulbar), ALSAQ-40, manual muscle testing (MMT), pinch strength and grip strength were performed.

### Outcomes

The BRC and placebo identities were revealed once the dosing was completed and we evaluated efficacy by two statistical analyses. The 1^st^ endpoint analysis compared baseline scores at the end of the observation period to those upon completion of stepwise dosing of 14 weeks (Fig 2). The 2^nd^ endpoint analysis compared the baseline score at the observation period endpoint to that at the time of study completion (Fig 2).

### Primary efficacy analysis I (ANCOVA)

Efficacy was estimated by a comparison of BRC and placebo groups mean scores at 1^st^ and 2^nd^ endpoints. Analysis of covariance (ANCOVA) was applied (p<0.05).

### Primary efficacy analysis II

The primary efficacy analysis II was a comparison of the mean of time-course score changes in BRC and placebo groups.

Efficacy and marginal significance were derived from a p-value of either primary efficacy analysis I (ANCOVA) or primary efficacy analysis II. Marginal significance was defined as a P-value within two-tailed test at 80% confidence level. Moreover, the time-course of the values of summary statistics, mean changes from baseline, and the slope of changes were also used in the analysis.

### Laboratory data

WBC count with fractionation, liver, and renal function tests as well as blood sugar were measured at regular intervals; electrocardiograms, echocardiograms, chest X-ray and vital signs were also taken.

### Statistical analysis

The study was sufficiently powered to independently assess a potential benefit of BRC compared with placebo for ALSFRS-R total scores and survival. Failure analyses undertaken for ALSFRS-R used the change from baseline to 1^st^ and 2^nd^ endpoints with the last observation carried forward (LOCF) for subjects who discontinued in Part I and Part II. We believe that the LOCF method was appropriate for exploratory analyses because of the very small number of drop-outs and the absence of study entrant deaths. The statistical analysis of this study was conducted by CMIC (CRO/CMO company, Statistical Analysis Division, Tokyo, Japan).

## Results

### Primary efficacy analysis I (ANCOVA)

**Fig 3:**
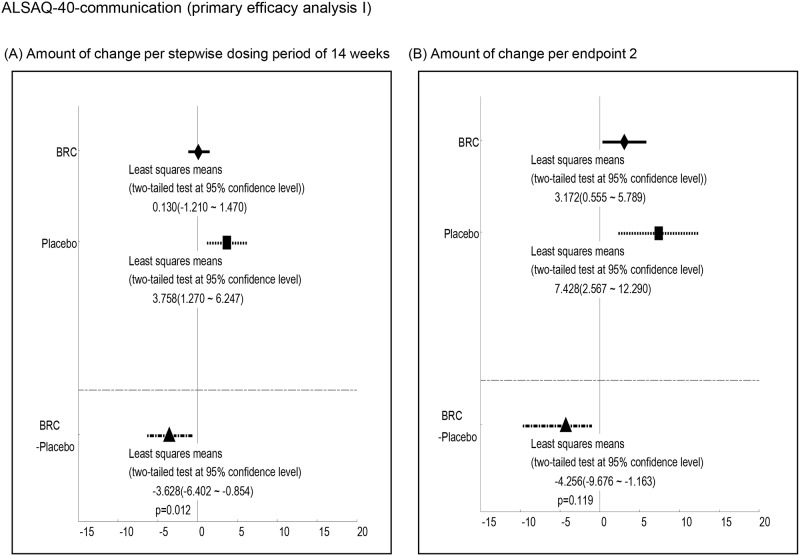
The results of primary efficacy analysis I on ALSAQ40 communication. In both 1^st^ endpoint and 2^nd^ endpoint analyses, the groups of BRC treatment were significantly recovered in ALSAQ40 communication compared to the groups of placebo (p < 0.2).

**Fig 4:**
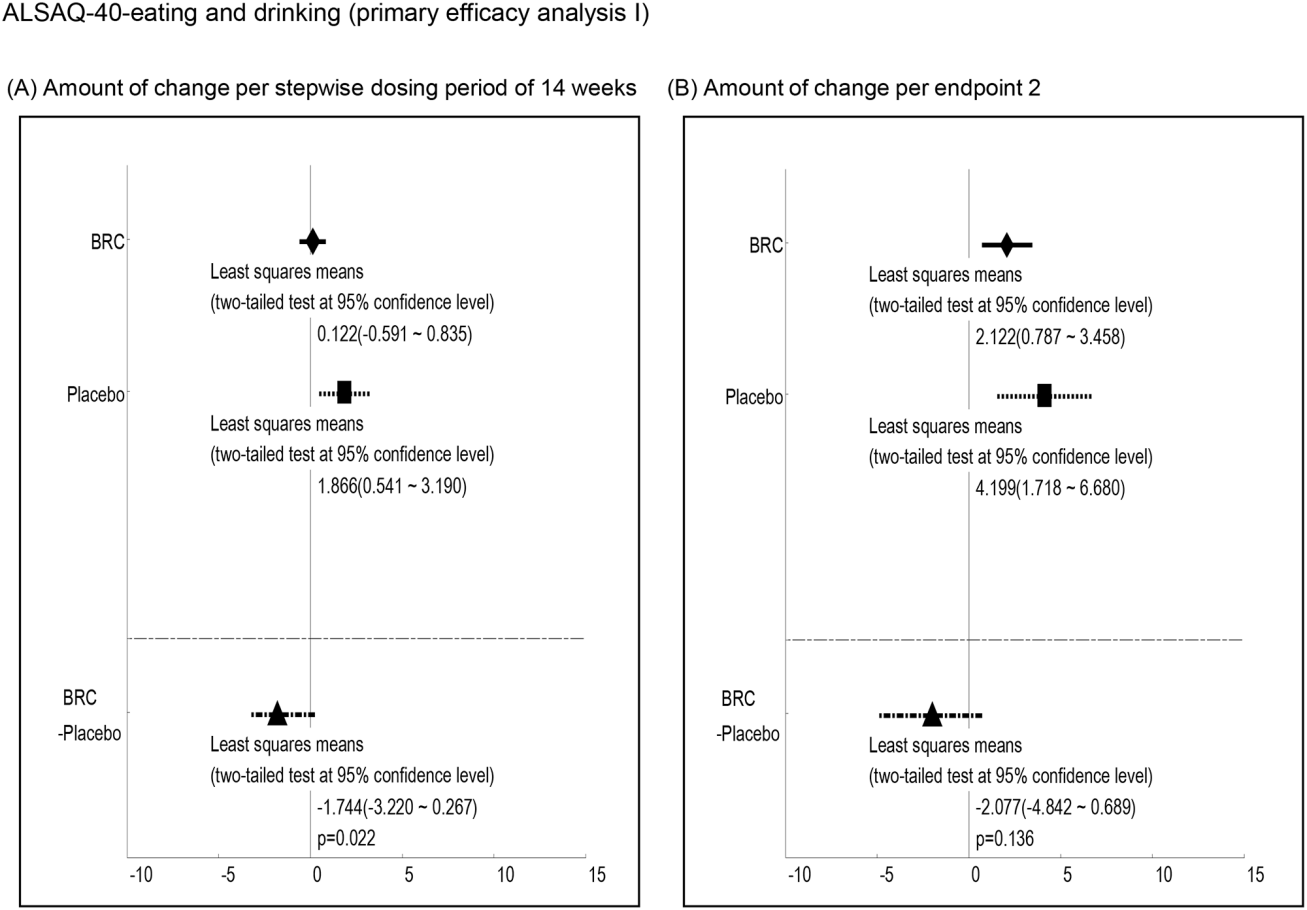
The results of primary efficacy analysis I on ALSAQ40 eating and drinking. In both 1^st^ endpoint and 2^nd^ endpoint analyses, the groups of NDDPX08 treatment were significantly recovered in ALSAQ40 eating and drinking compared to the groups of placebo (p < 0.2).

**Fig 5:**
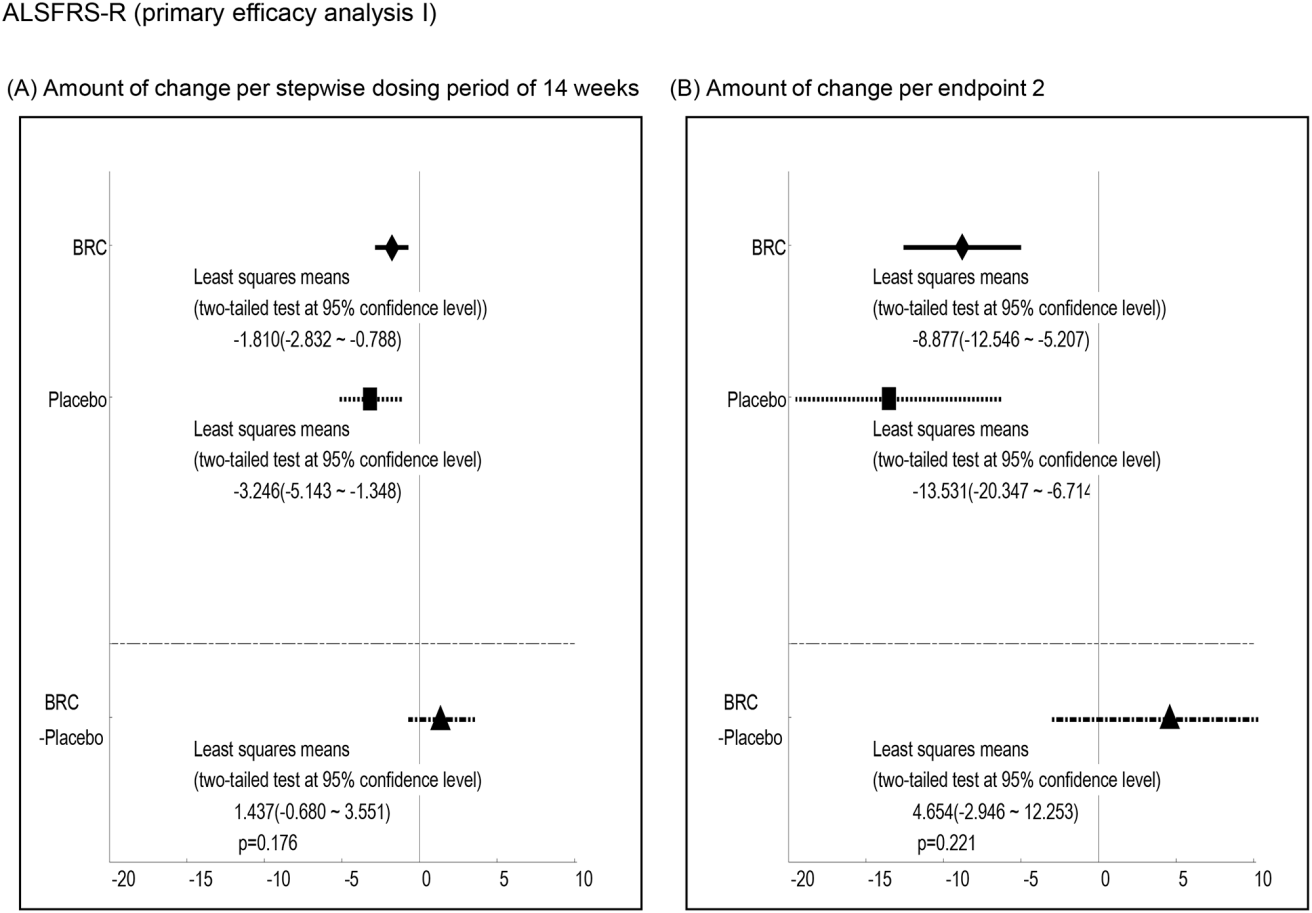
The results of primary efficacy analysis I on ALSFR-S. In 1^st^ endpoint analysis, the group of BRC treatment was significantly recovered compared to the group of placebo treatment (p < 0.2). Moreover, in 2^nd^ endpoint analysis, the group of BRC tended to be recovered in ALSFR-S total score compared to the group of placebo.

**Fig 6:**
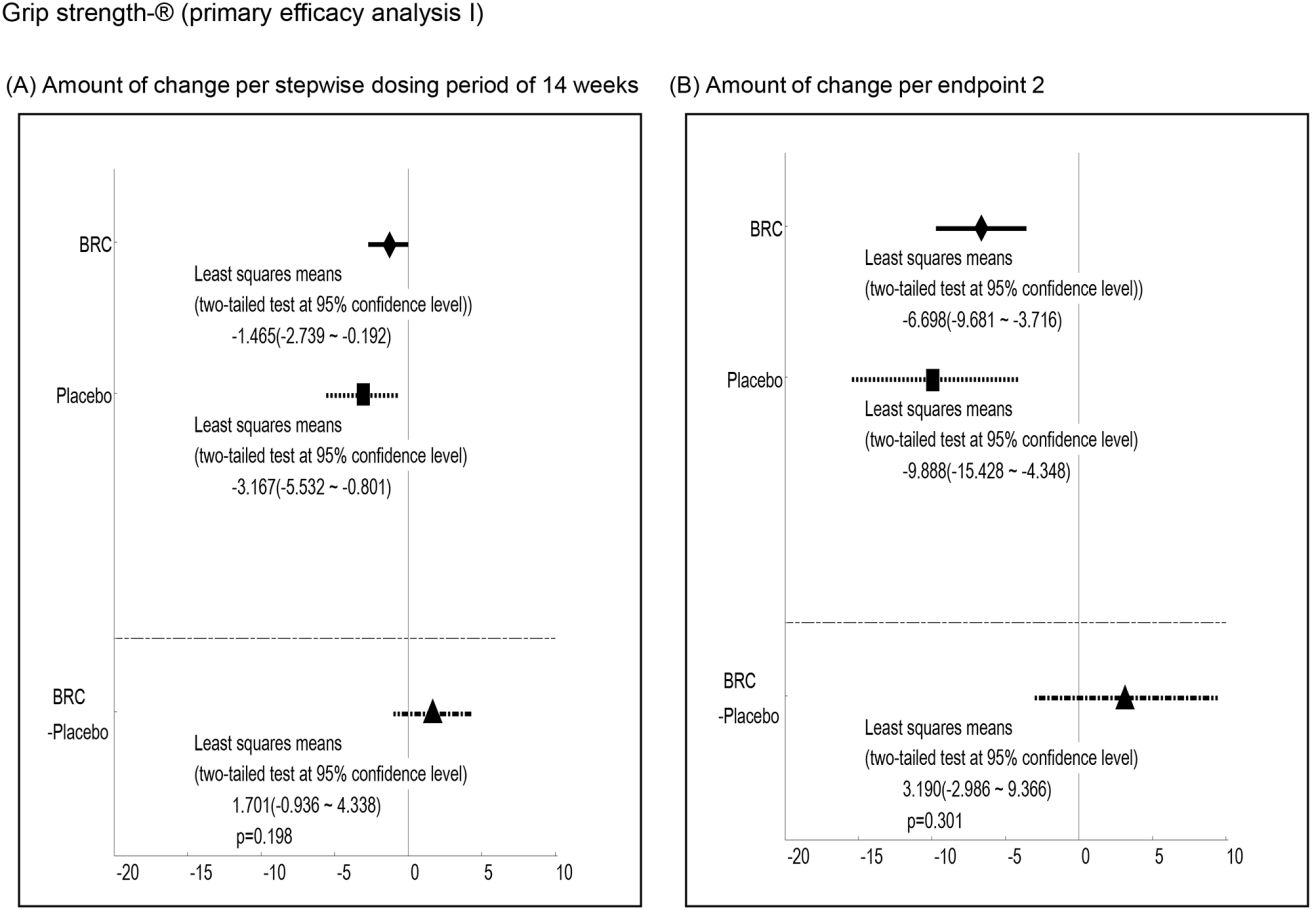
The results of primary efficacy analysis I on Grip strength (right side). In 1^st^ endpoint analysis, the group of BRC treatment was significantly recovered compared to the group of placebo treatment (p < 0.2). Moreover, in 2^nd^ endpoint analysis, the group of BRC tended to be recovered compared to the group of placebo.

**Fig 7:**
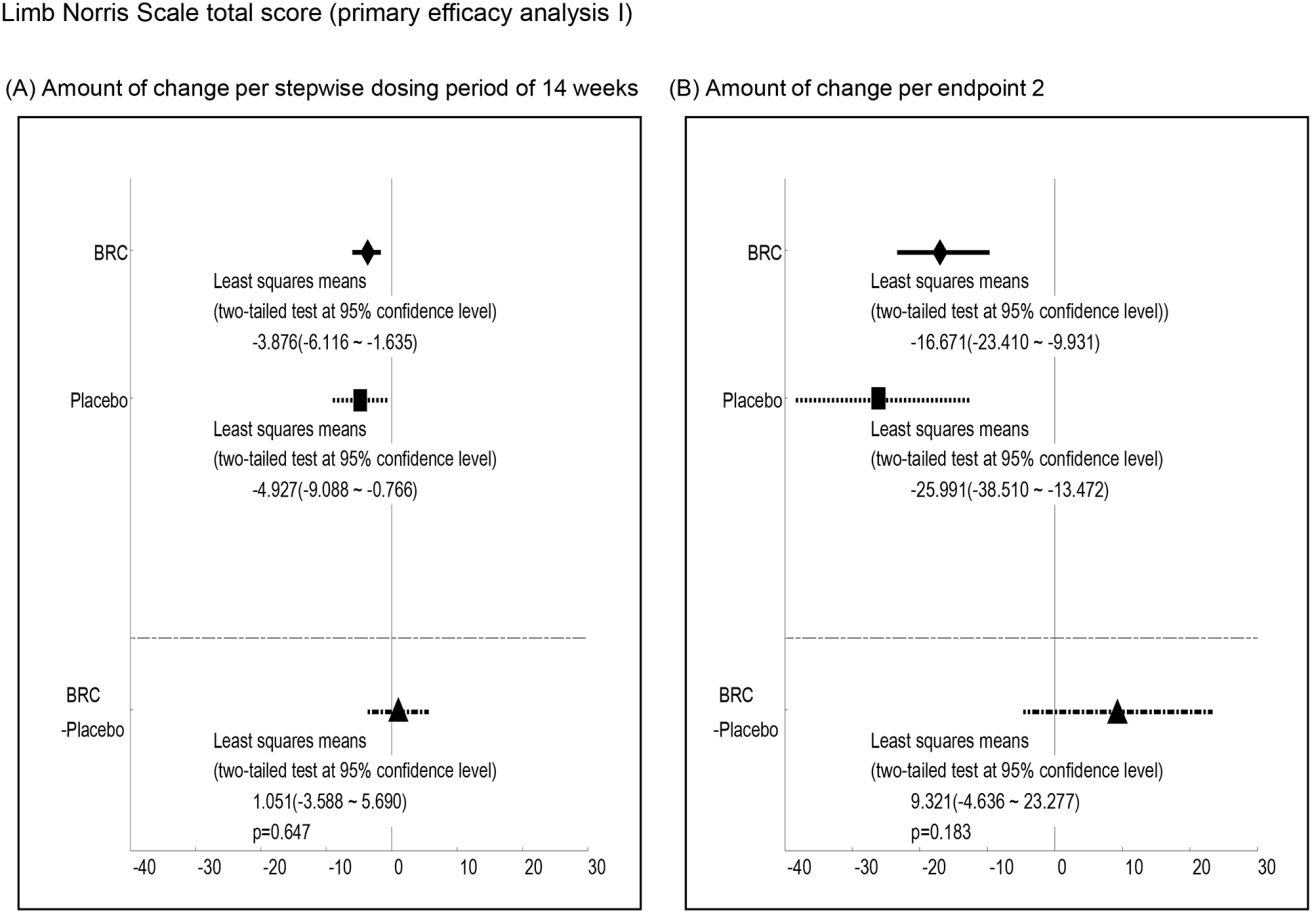
The results of primary efficacy analysis I on Limb Norris Scale total score. In 1^st^ endpoint analysis, the group of BRC treatment tended to be recovered compared to the group of placebo treatment. Moreover, in 2^nd^ endpoint analysis, the group of BRC was significantly recovered in Limb Norris Scale total score compared to the group of placebo (p < 0.2).

**Fig 8:**
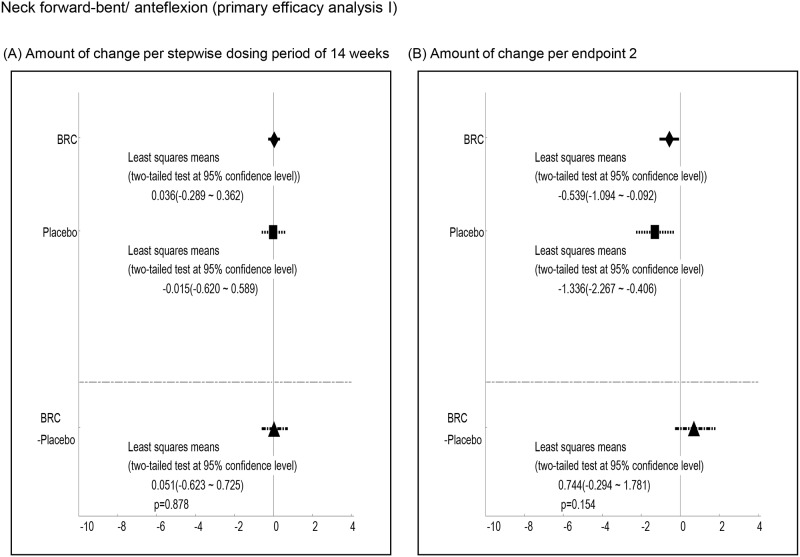
The results of primary efficacy analysis I on neck forward-bent/ anteflexion. In 1^st^ endpoint analysis, the group of BRC treatment tended to be recovered compared to the group of placebo treatment. Moreover, in 2^nd^ endpoint analysis, the group of BRC was significantly recovered compared to the group of placebo (p < 0.2).

Efficacy was analyzed by a comparison of the mean changes calculated at 1^st^ and 2^nd^ endpoints between BRC and placebo groups. Observed differences with P values less than 0.2 at the 1st endpoint included ALSAQ40-communication (P = 0.012, Fig 3(A)), ALSAQ40 eating & drinking (P = 0.022, Fig 4(A)), ALSFRS-R total score (P = 0.176, Fig 5(A)), and grip strength (right hand)(P = 0.198, Fig 6(A)). At the 2^nd^ endpoint analysis, significant differences were observed for Limb Norris Scale (P = 0.183, Fig 7(B)), ALSAQ40-communication (P = 0.119, Fig 3(B)), ALSAQ40 eating & drinking (P = 0.136, Fig 4(B)), and degree of neck flexion (P = 0.154, Fig 8(B)).

**Fig 9:**
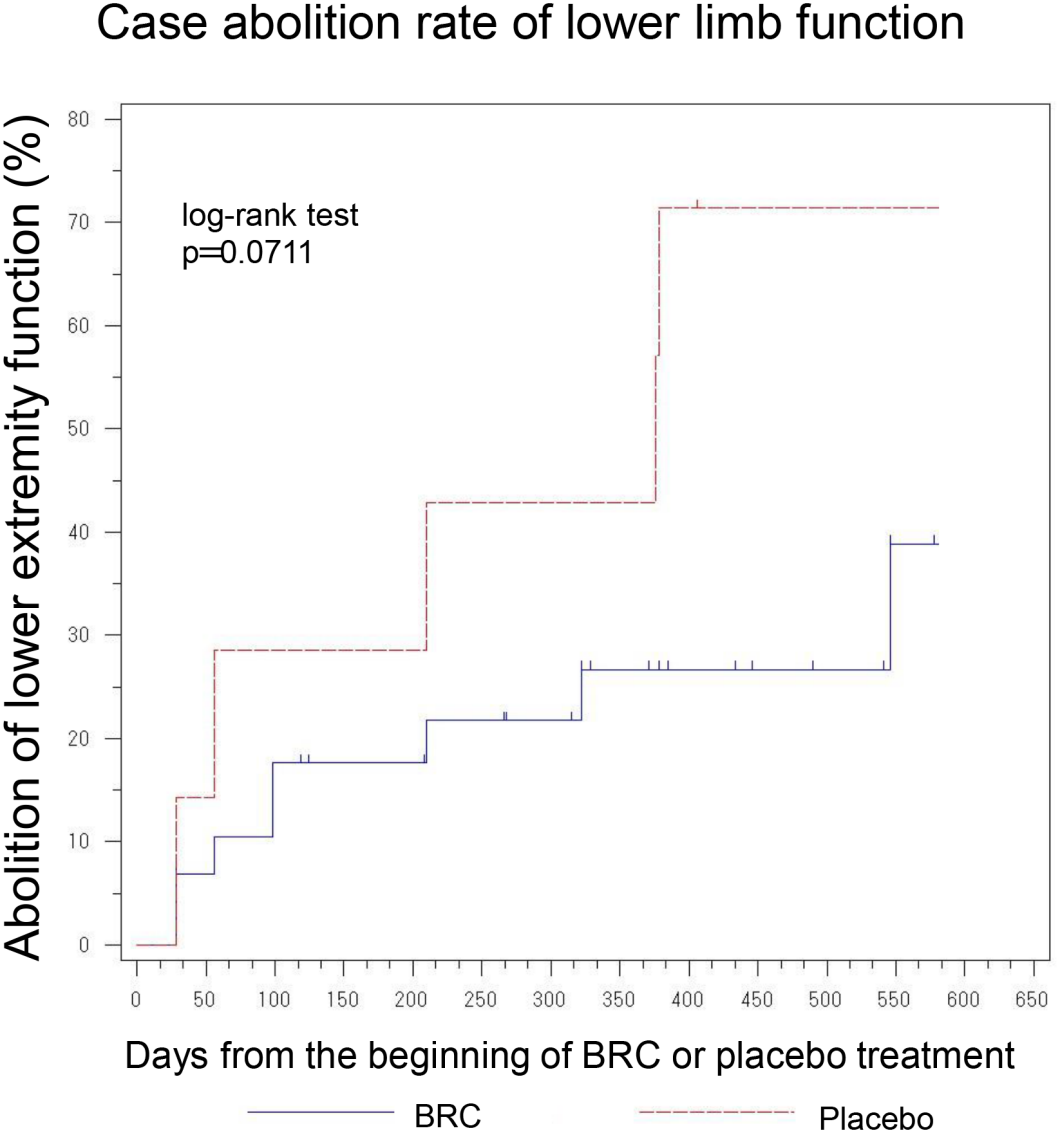
Case abolition rate of lower limb function. BRC signifiantly suppressed abolition rate of lower limb function in ALS compared to placebo.

Primary efficacy analysis II for the comparison of the mean of time-course score changes in BRC and placebo. P values of less than 0.2 were observed for the following items; Limb Norris Scale, ALSAQ40-communication, ALSAQ40 eating & drinking, and grip strength (right hand). Moreover, the abolition rate of lower limb function in ALS treated with BRC was less than that for non-treated ALS patients (Fig 9).

The above ALS scores indicated ALS severity was improved in BRC group compared to Placebo group.

On the other hand, almost patients were diagnosed with “definite” according to ALS diagnosis criteria. Moreover, there was no significant correlation between the efficacy for BRC and ALS progression.

### Safety (Table 2)

**Table 2:** Summary of safety assessment.

Adverse events	BRC (n = 31)	Placebo (n = 7)
Infection and infestations	19 (61.3%)	3 (42.9%)
(nasopharyngitis)	(11 (35.5%))	(1 (14.3%))
Gastrointestinal disorders	14 (45.2%)	4 (57.1%)
(constipation)	(5 (16.1%))	(2 (28.6%))
(nausea)	(5 (16.1%))	(2 (28.6%))
Skin and subcutaneous tissue disorders	12 (38.7%)	1 (14.3%)
(eczema)	(4 (12.9%))	(0 (0%))
Adverse drug events		
Gastrointestinal disorders	6 (19.4%)	3 (42.9%)
(nausea)	3 (9.7%)	2 (28.6%)
Serious adverse events		
Respiratory, thoracic and mediastinal	3 (9.7%)	0 (0%)
(aspiration pneumonias)	(2 (6.5%))	(0 (0%))
Serious adverse drug events		
Gastrointestinal disorder (ischemic colitis)	1 (3.2%)	0 (0%)

1. Adverse events
  The sole significant differences between the treatment groups were for comparatively minor issues including 19 infections, 14 gastrointestinal disorders, 12 skin and subcutaneous tissue disorders, 11 cases of nasopharyngitis, 5 individuals with constipations, 5 with nausea, and 4 with eczema in BRC group.
2. Adverse drug reactions
  There was no significant difference in incidence between the treatment groups expect for 6 gastrointestinal disorders and 3 nauseas in BRC group.
3. Serious adverse events
  There was no significant difference in serious adverse event incidence between the treatment groups. Three respiratory, thoracic and mediastinal disorders and 2 aspiration pneumonias according to the worse of ALS symptoms in both the treatment groups.
4. Serious adverse drug reactions
  There was no significant difference in serious adverse drug reactions incidence between the treatment groups; there was one case of gastrointestinal disorder and ischemic colitis.

### Laboratory data

**Fig 10:**
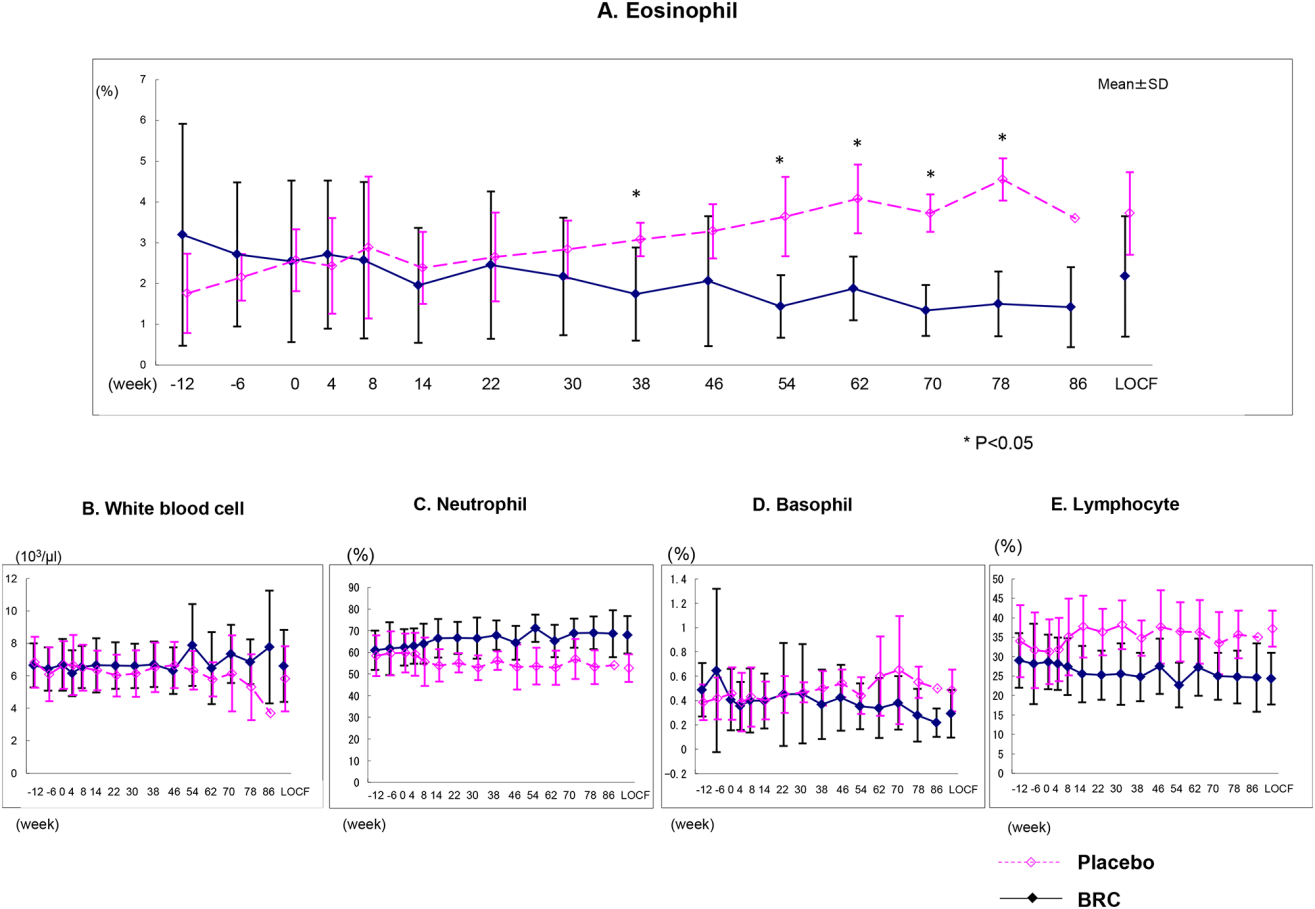
Alterations of differential white blood counts in ALS patients with or without BRC. Placebo treated ALS patients revealed significant increase of eosinophil. On the other hand, BRC treated ALS patients showed no increase of it (A). However, the number of the other blood cells stayed constant during the disease progression in both placebo and BRC treated group (B, C, D, E). LOCF: last observation carried forward. *P<0.05 BRC group vs Placebo group.

All laboratory data were within normal range with the exception of the increasing eosinophils exclusively seen in placebo treated ALS patients (Fig 10).

## Discussion

There are a limited number of therapeutic strategies that effectively relieve symptoms and improve the quality of life for ALS patients. Preclinical animal studies are an essential step on the path novel and effective therapeutic agents for the ALS. However, the outcomes of drug efficacy tests using animal models vary due to different methodological conditions such as the differences in giving pre-symptomatic versus post-symptomatic administration of agent [20]. In this regard, standard operating procedures for the preclinical animal study for ALS/MND has recently been published and strongly recommends the exclusive post-onset administration of candidate agents to approximate the clinical reality [21]. Previous studies have shown that dexpramipexole, a dopamine D2 receptor agonists used for Parkinson disease therapy might have the efficacy as an ALS treatment. Dexpramipexole is thought to enhance mitochondrial function and leads to increased survival and retention of motor function in *in vivo* models of ALS. Subsequently, EMPOWER, a phase 3 trial has shown that dexpramipexole although generally well tolerated did not differ from placebo in any prespecified efficacy endpoint measurement [22,23]. BRC is a unique member of the class of dopamine D2 receptor agonists with *in vitro* antioxidant properties [24–26]. Our previous studies have also shown that BRC upregulates NAIP and protects neuronal cells against oxidative insults independent of D2 receptor function as outlined below as well as delayings disease progression of ALS mouse. Interestingly, dopamine receptor antagonists (SCH23390: D1 dopamine receptor antagonist, Sulpiride: D2&D3 dopamine receptor antagonist, Raclopride: D2 dopamine receptor antagonist) did not affect BRC dependent anti-oxidative stress activity [27]. Interestingly, the alleviation of motor neuronal dysfunction in ALS mice by a post-onset administration of BRC was unchangeable by the presence or absence of Riluzole [27]. Furthermore, the results of this study imply that BRC in the presence of Riluzole is safe and well tolerated. Although this study had a comparative low number of ALS patients for an effectiveness evaluation, our results showed the BRC group showed trend toward efficacy suggesting it could be an effective treatment for ALS. In both primary efficacy analyses I and II, the patients treated with BRC had greater residual functions of the upper and lower limbs as reflected in the ALSAQ40-communication, ALSAQ40-eating & drinking, and Grip strength scores when compared to placebos (Figs 3, 4 and 6). Moreover, total ALSFRS-R scores and neck flexion test also tended to stability (Figs 5 and 8). The patients with early stage ALS showed the greatest effect on clinical scores. ALS is a complex neuromuscular degenerative disease, with an as yet undelineated and likely therapeutically refractory molecular pathology; the expectation of a panacea must be viewed as low. In this regard BRC protects cells against oxidative stress, which may underlie neuronal dysfunction (and/or degeneration) and manifestation of ALS but might not indeed is unlikely to account for all aspects of pathogenesis. It is conceivable for example that BRC sustains motoneuronal function at least in part through suppression of oxidative stress. BRC sustained both ALSFRS-R and grip strength until the 1^st^ endpoint but not the 2^nd^. On the other hand, BRC treated cases revealed some preservation of the Limb Norris and degree of neck flextion with 15mg/day dosing at the 2^nd^ endpoint. In addition placebo treated cases showed progressive deterioration of lower limb muscle function which BRC slowed (Fig 9). In addition to these encouraging functional effects, the progressive eosinophilia in the placebo but not BRC group was noteworth (Fig 10). This is first report of the cumulative increase of eosinophil but not other blood cells including basophil, neutrophil, and lymphocyte with ALS disease progression. Although the role of eosinophils in ALS is not clear, an upregulation of eosinophil-derived neurotoxin (END) from ALS patients but not Alzheimer or Parkinson diseases patients has been reported [28]. This may be attributable to neuronal inflammation via induction of oxidative stress. Whether eosinophil can be used as a biomarker for ALS, will emerge from closer analysis of the relationship between the eosinophil including END and ALS progression. In conclusion, the efficacy and safety of BRC should be further explored by further investigation of phase 2b/3 clinical trial.

## Supporting Information

S1 CONSORT Checklist (DOC)

S1 Protocol IRB protocol approved for the trial. (PDF)
