# Correction: Bromocriptine Mesylate Attenuates Amyotrophic Lateral Sclerosis: A Phase 2a, Randomized, Double-Blind, Placebo-Controlled Research in Japanese Patients

**DOI:** 10.1371/journal.pone.0152845

**Published:** 2016-03-28

**Authors:** Eiichiro Nagata, Mieko Ogino, Kounosuke Iwamoto, Yasuhisa Kitagawa, Yasuo Iwasaki, Fumihito Yoshii, Joh-E. Ikeda, ALS Consortium Investigators

## Notice of Republication

This article was republished on March 14, 2016, to correct the captions of Figs 3–7 and Figs 9–10 and figure citations in the Discussion section. Please download this article again to view the correct version. The originally published, uncorrected article and the republished, corrected articles are provided here for reference.

## Supporting Information

S1 File Originally published, uncorrected article. (PDF)

S2 File Republished, corrected article. (PDF)
