# Supplementary material for: Bromocriptine Mesylate Attenuates Amyotrophic Lateral Sclerosis: A Phase 2a, Randomized, Double-Blind, Placebo-Controlled Research in Japanese Patients
Source: PLoS One. 2016 Feb 24;11(2):e0149509. doi: 10.1371/journal.pone.0149509 (PMC4765990; doi:10.1371/journal.pone.0149509)
Supplement: S1 Protocol — (PDF) [file pone.0149509.s002.pdf]

Study on efficacy and safety of NDDPX08 in ALS patients

Research Organization

Tokai University Hospital

Representative Researcher: Joh-E Ikeda
Address: 143 Shimokasuya, Isehara 259-1193, Japan
Phone: 0463-93-1121 (ext. 2566)
FAX: 0463-91-4993
E-mail Address: jeikeda3@is.icc.u-tokai.ac.jp

November 18, 2008 (Version 1)
Revised February 6, 2009 (Version 1 1)
Revised April 9, 2009 (Version 1.2)
Revised April 22, 2009 (Version 1.3)
Revised October 20, 2009 (Version 2)

Confidentiality Policy

This protocol is treated as confidential information disclosed only to the parties involved in this clinical study, as listed below:

- **Institutional Review Board**
- **Study Director**
- **Investigator**
- **Sub-Investigator**
- **Study Contributors**
- **Director, Tokai University School of Medicine**
- **Director, Tokai University Hospital**
- **Units of Tokai University School of Medicine and Tokai University Hospital involved in this clinical study.**

Study Outline

1. Objectives

Amyotrophic lateral sclerosis (ALS) is a motor neuron disease involving selective impairment of motor nerves and presenting with progressive muscular weakness and atrophy. Its etiology remains to be clarified, and no drug adequately effective against this disease is available at present. Thus, it has been desired to develop new methods of treatment for this disease. In our previous studies using a system of cultured cells exposed to oxidative stress, it was shown that NDDPX08 (a compound currently used clinically as a means of treating Parkinson's disease) suppresses loss of nerve cell viability. Furthermore, treatment with NDDPX08 after the onset of ALS was shown to improve motor function and extend the survival period of transgenic mice (mice transfected with the familial ALS type 1 mutant SOD-1 gene, an animal model of ALS).

The present study is designed to evaluate the efficacy of NDDPX08 on motor function and quality of life (QOL) as well as its safety in patients with solitary or familial ALS.

2. Subjects

Patients diagnosed as having ALS and satisfying both requirement 1) and 2) are eligible for this study.

1) Patients in whom ALS is "definite, " "probable" or "probable-laboratory-supported" according to the EL Escorial Modified Airlie House Diagnostic Criteria and the severity is rated at grade 1, 2 or 3 according to the Ministry of Health, Labour and Welfare Criteria for ALS Severity.
2) Patients having given informed consent in writing to participate in the study.

3. Methods

Of the clinical study period lasting for 70-102 weeks, the first 12-week observation period involves treatment with Rilutek (generic name: riluzole) already approved as a drug for treatment of ALS in Japan (100 mg/day). Rilutek is initiated either at or 4 weeks before the start of the observation period. At the end of the observation period, the eligibility of each patient to participate in the study is checked again, and patients rated as eligible begin to receive treatment with a combination of NDDPX08 and Rilutek (combined treatment) according to an escalating dosing schedule (Fig. 1). During the first 2 weeks of combined treatment, the NDDPX08 dose level is set at 1.25 mg/day. Then, the NDDPX08 dose level is increased by 2.5 mg/day at intervals of 2 weeks, reaching 15 mg/day 12 weeks after the start of treatment. The NDDPX08 treatment period is 54-86 weeks. Treatment within the framework of this clinical study is deemed to have been completed at any of the following points of time: (1) beginning of 24-hour use of noninvasive respiration assistive device daily, (2) beginning of the use of an invasive respiration assistive device or (3) death of the patient.

The variables of efficacy and safety, listed below, are evaluated at the beginning of the observation period and at the start, during and the end of combined treatment. Changes in each variable are compared and analyzed (Fig. 2).

【Efficacy variables】 ALSFRS-R, %FVC, Modified Norris Scale, ALSAQ-40, MMT, pinching power, grip, ALS severity grade

【Safety variables】 Adverse events (particularly hallucination, nausea, vomiting, anorexia, etc.), blood pressure, heart rate, laboratory parameters, echocardiography findings

4. Study period and planned number of subjects

Study period: January 1, 2009 through January 31, 2011
Planned number of subjects: 50 cases (total number of patients entering the treatment phase)

5. Participating facilities

Department of Neurology, Tokai University School of Medicine (Main Hospital, Hachioji Hospital, Oiso Hospital)
Department of Neurology, Toho University School of Medicine (Omori Hospital)
Department of Neurology, Kitasato University School of Medicine (East Hospital)

Table of Contents

1. Objectives 6

2. Study organization 6

3. Background 8

4. Ethical considerations 8

5. Selection of subjects 10

6. Study period and planned number of subjects 11

7 Registration 11

8. Methods 12

9. Evaluations/tests and their schedules 17

10. Predictable adverse events 19

11. Handling of adverse events 20

12. Payment of expenses and compensation 21

13. Statistical analysis (Efficacy evaluation) 22

14. Publication of results 25

15. References 25

1. Objectives

On the basis of the findings from our basic studies conducted to date, the present study is undertaken to evaluate the efficacy of orally administered NDDPX08 (a drug currently used clinically for the treatment of Parkinson's disease) on motor function and quality of life (QOL) as well as its safety in patients with solitary or familial amyotrophic lateral sclerosis (ALS).

2. Study organization

1) Study Director

Joh-E Ikeda, Professor, Division of Basic Medicine, Tokai University School of Medicine

2) Researchers

Shigeharu Takagi, Professor, Division of Internal Medicine, Tokai University School of Medicine (Department of Neurology, Tokai University Hospital); in charge of implementing the clinical study and evaluating safety

Fumihito Yoshii, Professor, Division of Internal Medicine, Tokai University School of Medicine (Department of Neurology, Tokai University Hospital); in charge of implementing the clinical study and evaluating safety

Shunya Takizawa, Professor, Division of Internal Medicine, Tokai University School of Medicine (Department of Neurology, Tokai University Hospital); in charge of implementing the clinical study and evaluating safety

Eiichiro Nagata, Lecturer, Division of Internal Medicine, Tokai University School of Medicine (Department of Neurology, Tokai University Hospital); in charge of implementing the clinical study and evaluating safety

Tatsuya Ishikawa, Research Associate, Division of Internal Medicine, Tokai University School of Medicine (Department of Neurology, Tokai University Hospital); in charge of implementing the clinical study and evaluating safety

Yasuhisa Kitagawa, Professor, Division of Internal Medicine, Tokai University School of Medicine (Department of Neurology, Tokai University Hachioji Hospital); in charge of implementing the clinical study and evaluating safety

Kaytsunori Akiyama, Lecturer, Division of Internal Medicine, Tokai University School of Medicine (Department of Neurology, Tokai University Oiso Hospital); in charge of implementing the clinical study and evaluating safety

Yasuo Iwasaki, Professor, Department of Neurology, Toho University Omori Medical Center; in charge of implementing the clinical study and evaluating safety

Mieko Ogino, Lecturer, Department of Neurology, Kitasato University School of Medicine (Department of Neurology, Kitasato University East Hospital); in charge of implementing the clinical study and evaluating safety

3) Study Contributors

Hirohide Takahashi, Associate Professor, Division of Internal Medicine, Tokai University School of Medicine; in charge of evaluating safety

Wakao Takahashi, Associate Professor, Division of Internal Medicine, Tokai University School of Medicine; in charge of evaluating safety

Yuko Onuki, Research Associate, Division of Internal Medicine, Tokai University School of Medicine; in charge of evaluating safety

Kyuichiro Onoue, Lecturer (Part Time), General Institute of Medicine, Tokai University; in charge of evaluating safety

4) Investigator (nominated if Study Director is not a physician)

Fumihito Yoshii, Division of Internal Medicine, Tokai University School of Medicine (Department of Neurology, Tokai University Hospital)

5) Clinical Study Coordinator

·Joh-E Ikeda, Neurodegenerative Disease Research Centre, Tokai University Graduate School of Medicine; in charge of coordinating the study and managing the information in collaboration with the participating facilities and CRC

6) Drug Administrator

Kun Ichikawa, Director of Pharmacy, Pharmacy Division, Tokai University Hospital; in charge of adjusting and managing drugs

7) Participating facilities

Department of Neurology, Tokai University Hospital
Department of Neurology, Tokai University Oiso Hospital
Department of Neurology, Tokai University Hachioji Hospital
Department of Neurology, Toho University Omori Medical Center
Department of Neurology, Kitasato University East Hospital

8) ALS Treatment Plan Evaluation Committee

In charge of supervising and encouraging implementation of this clinical study (a multicenter study) and evaluating and judging the efficacy and safety of NDDPX08 at the end of the study

Chairman

Ichiro Kanazawa, Chief Medical Officer, Grand Steward's Secretariat, Imperial Household Agency; Professor, International University of Health and Welfare Graduate School

Members

Shigeki Katsurahara, Director, National Center of Neurology and Psychiatry
Yasuto Itoyama, Professor, Department of Neurology, Tohoku University School of Medicine
Hajime Sofue, Professor, Department of Neurology, Nagoya University School of Medicine

9) Secretariat for Clinical Study on ALS (FeGALS Office)

Neurodegenerative Disease Research Centre, Department of Frontier Medicine, Tokai University Graduate School, 143 Shimokasuya, Isehara, Kanagawa 259-1193, Japan

TEL: 0463-93-1121(switchboard) Ext. 2568 FAX: 0463-91-4993 (direct)

3. Background

Amyotrophic lateral sclerosis (ALS) is a motor neuron disease involving selective impairment of motor nerves and presenting with progressive muscular weakness and atrophy. Its etiology remains to be clarified, and no drug adequately effective against this disease is available at present. Thus, it has been desired to develop new methods of treatment for this disease. In our previous studies using a system of cultured cells exposed to oxidative stress, it was shown that NDDPX08 (a compound currently used clinically as a means of treating Parkinson's disease) suppresses loss of nerve cell viability. Furthermore, treatment with NDDPX08 after the onset of ALS was shown to improve motor function and extend the survival period of transgenic mice (mice transfected with the familial ALS type 1 mutant SOD-1 gene, an animal model of ALS).

The present study is designed to evaluate the efficacy of orally administered NDDPX08 on the motor function and quality of life (QOL) as well as its safety in patients with solitary or familial ALS. NDDPX08 has been used for 23 years since approval as a drug for the treatment of Parkinson's disease, and adequate data are available on its efficacy and safety when used for such a purpose.

4. Ethical considerations

1) Compliance with ethical guidelines related to clinical studies

This clinical study is carried out in accordance with the ethical principles set forth in the "Ethical Guidelines on Clinical Studies (enforced on April 1, 2005)" prepared by the Ministry of Health, Labour and Welfare (MHLW), the "Rules and Bylaws on Clinical Studies at Hospitals Attached to Tokai University School of Medicine" and the protocol for this study.

2) Ethics Committee

This clinical study needs to be approved in advance by the Institutional Review Board of the Tokai University Hospital.

The Study Director is required to submit a "Report on Status of Clinical Study" to the Institutional Review Board (IRB) at intervals of one year and to follow the view of the IRB as to the appropriateness of further continuing the study (if the study lasts more than one year). Within one month after completion or discontinuation of the study, the Study Director is required to submit a "Report on Completion (Discontinuation) of the Study" to the IRB. Upon the onset of an event of category (a) listed below, the report needs to be submitted within one week after said onset.

The Study Director is required to report any of the following events or cases to the IRB and to follow the view of IRB as to the appropriateness of further continuing the study.

(a) Onset of serious adverse events or the like

(b) Major modification of the protocol for this study
(c) Major modification of the informed consent form or other leaflets
(d) Modification of other documents subjected to IRB check
(e) Other cases judged by the Hospital Director as requiring IRB check

3) Acquisition of informed consent from subjects

Prior to the start of this study, the Investigator or Sub-Investigator informs each candidate patient of the following aspects of the study using the leaflet and obtains consent from the patient to participate in the study issued in writing at his/her own discretion.

The leaflet needs to include the following information:

(1) The research aspect of the treatment involved in the study;
(2) Objectives of the study;
(3) Methods of the study (explorative aspects of the study, patient inclusion criteria, etc.);
(4) Planned duration of participation in the study;
(5) Planned number of subjects of the study;
(6) Predicted clinical advantages, risks and inconveniences (if no clinical advantage is predicted, each patient needs to be so informed);
(7) Availability of other treatment methods for patients and predicted important advantages and risks of such treatment methods;
(8) Treatment which subjects of this study are entitled to receive upon onset of health hazards related to the study;
(9) The capability of each patient to decide whether or not to participate in the study at his/her own discretion; the capability of each patient or his/her proxy consenter to refuse or cancel participation in the study any time; and the policy that refusal or cancellation of participation in the study does not cause any disadvantage for the patient or cause the patient to suffer loss of benefits he/she is reasonably entitled to;
(10) The policy that the patient will be immediately informed of any information possibly affecting the intention of the subject or his/her proxy consenter to remain in the study, after acquisition by the subject or the proxy consenter;
(11) Criteria for rejection of patients from the study;
(12) The policy that the Monitor, Auditors, members of the IRB and domestic/overseas regulatory authority officials may be allowed access to the raw data (medical records, etc.) of the subject, thereby assuring that adequate measures will be taken to protect the privacy of the subject, and that signing of the informed consent form by the patient or the proxy consenter constitutes authorization of access to such data by these parties;
(13) The policy that privacy of the subject will be protected when the results of the study are made public;
(14) Details of the costs to be borne by the subject, if any;
(15) Name, title and contact address of Investigator/Sub-Investigator and Study Contributors (the name, title and contact address of Clinical Study Coordinator, if any);
(16) The unit of the medical facility to be contacted by the subject when he/she desires further information related to the study and his/her rights;
(17) Any responsibilities the subject must fulfill.

4) Supply of information to subjects

If information possibly affecting the intention of the subject or his/her proxy consenter to remain in the study has been obtained, the Investigator is required to furnish the subject or the proxy consenter with such information immediately and to confirm his/her intention to remain in the study. The steps taken on this topic need to be recorded in the medical records.

5) Protection of privacy

The privacy of each subject of this study is protected.

6) Protection and management of personal information

The personal information of the subjects of this study is protected by linkable anonymization. The information of all subjects of this study is managed collectively by the Personal Information Administrator. When the results of the study are published by means of presentation or papers, non-linkable anonymization is used to avoid identification of individual subjects.

Personal Information Administrator: Fumihito Yoshii, Professor, Division of Internal Medicine, Tokai University School of Medicine

5. Selection of subjects

1) Eligible patients

Patients diagnosed as having ALS and satisfying all of the following requirements are eligible for this study.

(1) Patients in whom ALS is "definite," "probable" or "probable-laboratory-supported" according to the EL Escorial Modified Airlie House Diagnostic Criteria (Attachment 1);
(2) Patients having received no treatment with NDDPX08 before;
(3) Patients whose ALS is rated as grade 1, 2 or 3 according to the ALS Severity Criteria (Attachment 2);
(4) Patients with %FVC of 70% or higher;
(5) Patients who developed ALS within 3 years before giving informed consent to participate in this study;
(6) Patients between 20 and 75 years of age at the time of giving informed consent in writing;
(7) Patients having given informed consent to participate in the study in writing (as a rule, the patient's own signature is needed, but the signature may be attached by the proxy consenter if the patient is unable to do so due to hand dysfunction);
(8) Patients with a magnitude of change in ALSFRS-R score (Attachment 3) between -1 and -4 during the pretreatment 12-weeek observation period.

2) Exclusion criteria

Patients falling under any of the following criteria (1) through (10) are excluded from the study.

(1) Patients to whom the diagnosis has not been disclosed;
(2) Patients with a history of allergy to any component of the test drug;
(3) Patients with severe psychiatric symptoms (hallucination, delusion) or dementia;
(4) Patients with severe orthostatic hypotension or other types of hypotension;
(5) Patients with a severe complication(s) such as heart, kidney and/or liver disease;
(6) Patients found by echocardiography to have valvular heart disease (e.g., valve hypertrophy,

restricted range of valve motion, stenosis associated with such abnormalities, etc.) or having a history of such disease (patients with moderate disease of only one valve are eligible for the study);

(7) Pregnant, possibly pregnant or lactating women;

(8) Patients using any other clinical trial drug or having participated in any other clinical trials within 3 months before;

(9) Patients incompetent to give consent to participate in the study;

(10) Other patients judged by the attending physician as inappropriate for the study.

3) Criteria for discontinuation

(1) Onset of serious adverse events or the like;

(2) Cancellation of consent to participate in the study by the patient or his/her proxy consenter;

(3) Cases where the attending physician recommends discontinuation of participation in the study;

(4) Other reasons requiring discontinuation.

【Rationale】

Exclusion criteria (1) through (10) and criteria for discontinuation (1) through (4) are adopted in view of the necessity to implement the study in a safe and ethically acceptable manner and to collect reliable data from the study.

6. Study period and planned number of subjects

Study period: January 1, 2009 through January 31, 2011

Planned number of subjects: 50 cases (total number of patients entering the treatment phase)

【Rationale】

In view of the explorative nature of this study, the minimum number of subjects sufficient to yield statistically significant differences, taking into account feasibility at the participating facilities, is adopted.

7. Registration

The Study Director registers patients as subjects of this study after confirming that they satisfy the inclusion criteria, fall under none of the exclusion criteria and plan to receive treatment with NDDPX08 for a period of at least 54 weeks during the study period.

Stated concretely, the following steps are taken for registration.

Temporary registration: The Researcher at a participating facility enters the information about the start of the observation period for each patient satisfying the above-mentioned criteria and having given informed consent in Form 1 (Temporary Registration Form; entry by CRC acceptable) and dispatches it by FAX to the FeGALS Office (Secretariat for Clinical Study on ALS). The FeGALS Office enters the patients in the Registration Control List in order of the time of arrival of the Temporary Registration Form (Form 1).

Formal registration: A Researcher at a participating facility evaluates each patient at the end of the observation period and checks whether or not the patient satisfies the requirements, using the Formal Registration Form (Form 2). The Researcher then enters necessary information into Form 2 and dispatches it by FAX to the FeGALS Office. The FeGALS Office assigns a drug allocation code to each

patient, using a table of random numbers, after receipt of the Formal Registration Form. The drug allocation code assigned is sent by FAX to the CRC in charge at the corresponding participating facility, using the Drug Allocation Notification Form (Form 3). In addition, the FeGALS Office enters the information about the start of treatment for each patient in the Registration Control List. The CRC receiving the Drug Allocation Notification Form requests the Researcher to prescribe the drug for the patient concerned, without notifying the Researcher of the drug allocation code for the patient. (The prescription ordering system at each participating facility needs to be arranged appropriately to ensure blinding of the drug/placebo prescribed for each subject of this study).

8. Methods

1) Multicenter double-blind study procedure

【Drug treatment】

Efficacy and safety are evaluated through analysis of changes in each variable measured at the start of the observation period and before and after the start of treatment with NDDPX08 or placebo.

For patients who begin to receive Rilutek treatment at the start or 4 weeks before the start of the observation period, the 12-week treatment with Rilutek alone (100 mg/day) during the observation period is followed by combined treatment (Rilutek + NDDPX08). The NDDPX08 dose level begins at 1.25 mg/day and is increased in steps to 15 mg/day during the 12-week treatment period according to the dose escalation schedule given on the next page (Fig. 1). If any serious adverse reaction arises following a dose increase to 10 mg/day and it is judged to be difficult to maintain this dose level, the dose level of 7.5 mg/day is regarded as the maintenance dose level. If alleviation of symptoms is noted during dose escalation steps, the dose level producing alleviation of symptoms is used as the maintenance dose level.

The total NDDPX08 treatment period is 58-90 weeks (including the 4 weeks during which the dose level is reduced in steps). Follow-up of adverse events is continued until 1 month after the end of NDDPX08 treatment.

【Allocation of placebo】

Placebo (lactose) is administered to 10 of the 50 subjects planned to be enrolled in the study. Patients registered as subjects of this study at 3 hospitals of Tokai University, Kitasato University East Hospital and Toho University Omori Medical Center are combined and numbered 1 through 50 in order of the time of registration.

Allocation of NDDPX08 is performed by the Tokai University Secretariat for Clinical Study on ALS (FeGALS Office), using a table of random numbers. The information on drug allocation is maintained securely as confidential information.

Fig. 1 Flow chart of the clinical study (drug treatment method): NDDPX08 dose escalation schedule

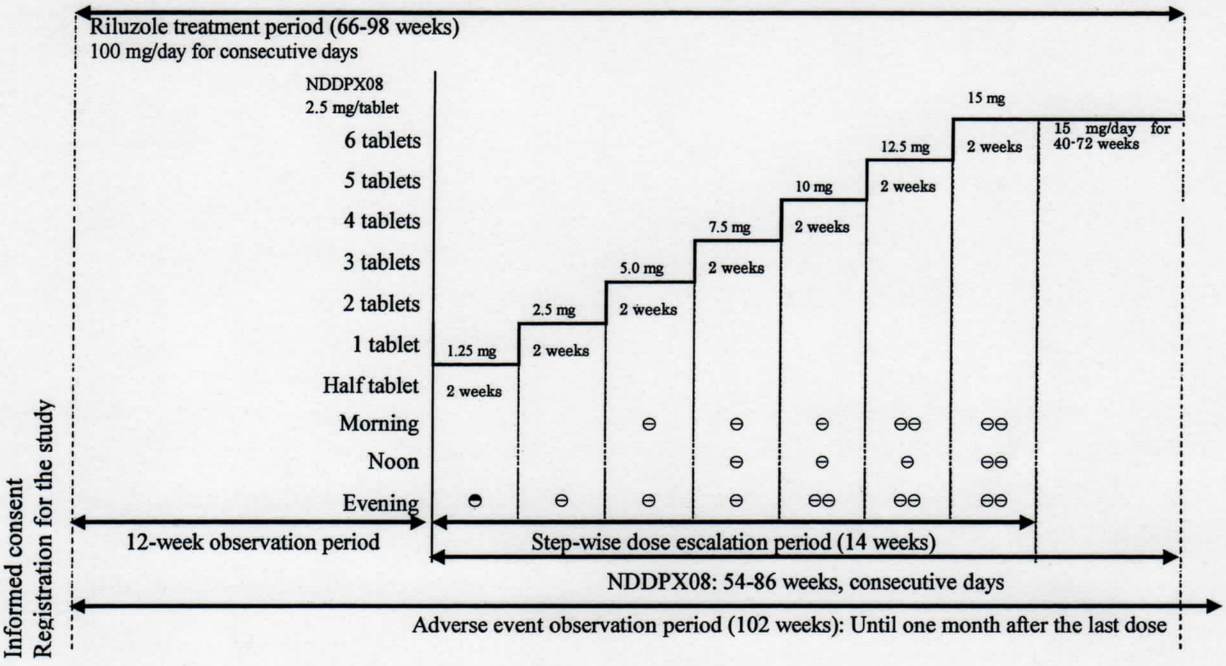

【Dispensing】

The placebo (lactose: 250 mg and 500 mg) and NDDPX08 2.5 mg tablet used for this study are in powder form. Each NDDPX08 2.5 mg tablet weighs 140 mg. To minimize the amount of drug remaining in the pack after use (to minimize errors in drug dose levels), the 140 mg powder equivalent to one NDDPX08 2.5 mg tablet is combined with 110 mg lactose to a total weight of 250 mg. If half of an NDDPX08 2.5 mg tablet (=1.25 mg tablet) is administered at a time, the powdered drug is combined with 180 mg lactose to a total weight of 250 mg. If two NDDPX08 2.5 mg tablets are administered at a time, the powdered drug (280 mg) is combined with 220 mg lactose to a total weight of 500 mg. Each active drug (powder equivalent to 1.25 mg, 2.5 mg and 5.0 mg of the active ingredient) and each placebo (powdered lactose 250 mg and 500 mg, equal in amount to the powdered active drug) are packed and stored shielded from light.

The placebo and active drug in powder form are prepared at the pharmacy of each participating facility (Tokai University Hachioji/Oiso Hospital, Kitasato University East Hospital, Toho University Omori Medical Center). The drug/placebo (NDDPX08 2.5 mg tablet, lactose) and materials (packing paper, light-shielding and other materials needed for dispensing of powder preparations) are supplied by the FeGALS Office. The manuals for powder dispensing, identification and control of the active drug and placebo, etc. are supplied by the Pharmacy of the Tokai University Hospital and the FeGALS Office CRC.

【Distinction between active drug and placebo】

A drug code No. (NDDPX08-TSM-specific 6-digit alphanumeric) is printed on the paper pack for NDDPX08. The times for drug intake (morning, noon and evening) and the amount of drug contained (1.25 mg, 2.5 mg and 5.0 mg) are printed on each pack. A drug code No. (NDDPX08-TSM-specific 6-digit alphanumeric) is also printed on the paper pack for the placebo (lactose). The times for drug intake

(morning, noon and evening) and the amount of drug contained (1.25 mg, 2.5 mg and 5.0 mg) are printed on each pack. The active drug (NDDPX08) and its placebo are prepared at the pharmacy of each participating facility and subsequently stored and managed strictly. The Investigator delivers either the active drug or the placebo to each subject.

The drug code No. is composed of the test drug code (NDDPX08), the code for each participating facility (Tokai University Hospital = TSM, Tokai University Hachioji Hospital = TSH, Tokai University Oiso Hospital = TSO, Kitasato University East Hospital = KHH, Toho University Omori Medical Center = TMC) and the active drug/placebo code (identifiable specific 6-digit alphanumeric).

【Blinding protocol】

At the end of the 12-week observation period, the Investigator judges the eligibility of each temporarily registered patient in the treatment phase of the study. If rated as eligible, the patient is registered via FAX (formal registration), and the FeGALS Office assigns a drug allocation code No. (selected from a table of random numbers) to this patient on the basis of the formal registration form. Then, FeGALS dispatches the Drug Allocation Notification Form by FAX to the CRC of the participating facility concerned. The CRC receiving the Drug Allocation Notification Form transmits the information about the drug allocation code No. to the Pharmacy (or sends the drug allocation notification form directly by FAX). Each party involved checks the information and the CRC requests that the attending physician issues a drug prescription while keeping the physician uninformed as to the drug allocation code (i.e., whether the prescription is an active drug or a placebo). The Pharmacy delivers the drug of the corresponding drug allocation code No. to the patient after receiving the prescription issued by the attending physician. The CRC of each participating facility gives adequate accounts to the subject or his/her family as to the procedures for drug storage, drug intake and re-collection of the drug package used or kept unsealed.

The CRC of each participating facility re-collects the drug packages brought by the patient during hospital visits to check the status of compliance with dosing instructions. After temporary storage by this CRC, the re-collected drug package is handed to the CRC of the Tokai University ALS Clinical Study Group during the patient's visit.

The FeGALS Office stores all re-collected drug packages. Prior to statistical analysis of efficacy by an outside contractor, the drug remaining in the re-collected packages and the unused drug package (extracted at random) are subjected to identification by an outside testing facility and comparison to the drug allocation codes.

Prior to statistical analysis of the data on each subject, the FeGALS Office submits the medication (active drug/placebo) information as well as that about the drug remaining in the packages and the unused drug packages to the ALS Treatment Plan Evaluation Committee.

【Placebo allocation】

The placebo is allocated by dynamic allocation. Dynamic allocation involves the following two factors.

Factor 1 ALS severity grade (1, 2 and 3)

Factor 2: Age (60 years)

【Cooperation among CRCs of multiple participating facilities】

This clinical study on ALS is carried out at multiple facilities (Tokai University, Kitasato University and Toho University). To ensure smooth implementation of the clinical study, universal validity of the efficacy evaluation criteria and objectivity of evaluation, CRCs (clinical research coordinators) are incorporated in accordance with the clinical study protocol. The CRCs at all participating facilities

cooperate with each other to facilitate monitoring of the clinical study protocol, management of information on temporary and formal registration of subjects, management of patient visits, tests and drug intakes, monitoring of evaluations, management of evaluation-related information and smooth linkage among the attending physicians, evaluators (neurologists) and pharmacies. To this end, the CRC (University CRCs Cooperation) Secretariat is organized within the FeGALS Office, and the Tokai University CRC is nominated as the head of the CRC Secretariat. The CRCs of all participating facilities share the information about the hospital visit schedule, tests, questionnaire survey, medication protocol, etc. for each subject. The CRC Secretariat carries out intensive management of these pieces of personal information and maintains strict custody of such information. A CRC worksheet and CRF of uniform design are employed for this study (Cf. CRF list and sample forms attached). The CRC Secretariat delivers the CRC worksheet and CRF of uniform design to the CRC of each participating facility.

【ALS Treatment Plan Evaluation Committee】

The ALS Treatment Plan Evaluation Committee is organized as an outside unit of this study to ensure the reliability and subjectivity of study implementation and evaluation. The duties assigned to this committee are adjustment and encouragement of the study and evaluation and judgment of the safety and efficacy of NDDPX08 in patients with ALS at the end of the clinical study.

【Prohibited concomitant drugs】

Whether or not a concomitant drug or other drugs during the study period are approved is judged in accordance with the criteria given below. Concomitant use of the drugs listed below, which can affect evaluation of the efficacy of NDDPX08, is prohibited for the period from 4 weeks before the start of Rilutek until the end of the study period.

(1) Dopamine antagonists: antipsychotics such as metoclopramide and sulpiride
(2) CYP1A2 inhibitors: ciprofloxacin, enoxacin, fluvoxamine, etc.
(3) Estrogen preparations
(4) L-dopa preparations
(5) Dopamine agonists
(6) Anticholinergics
(7) Amantadine hydrochloride
(8) Droxidopa
(9) Selegiline hydrochloride
(10) COMT inhibitors
(11) Mecobalamin
(12) Edaravone

【Other restricted drugs】

(1) It is prohibited to begin treatment with any other drug during the period from 4 weeks before the start of the observation period until the end of the study. Temporary concomitant use of drugs is permitted if aimed at dealing with accidental complications, etc., but the use of such drugs needs to be minimized. (Example: cold medicine, analgesics, wet packs, drugs for pollinosis, etc.)
(2) Concomitant use of drugs is permitted if the drugs begin to be used 4 weeks or more before the start of observation and they are used without changing the dosing method or level. Throughout the study period, the dosing method and level of concomitant drugs must be kept unchanged.
(3) Temporary concomitant use of the antiemetic Nauzelin is permitted.

【Concomitant therapy】

The standards given below apply to concomitant therapy or the like during the study period.

(1) Exercise therapy such as rehabilitation

Exercise therapy is applied in a uniform manner as far as possible to all participating facilities. Excessive exercise, which can reduce the muscular strength, is avoided, and the level of exercise is kept equivalent to stretching or the like. Entry into the column "Concomitant therapy" of Form 10 is unnecessary.

(2) Other therapies

No other therapy is permitted, as a rule, except for cases where such therapy is needed to deal with progression of the disease. In cases where such therapy is used, details need to be entered into the column "Concomitant therapy" of Form 10.

2) Efficacy variables

a. Primary efficacy variable

・ALSFRS-R score (Attachment 3)

b. Secondary efficacy variables

・Length of time until death or disease progression to a certain stage (loss of unassisted gait capability, loss of arm function, tracheotomy, attachment of a respirator, tube feeding)
・%FVC
・Modified Norris Scale score (Attachment 4)
・ALSAQ-40 score (Attachment 5)
・MMT (manual muscle test)
・Pinching power
・Grip
・ALS severity grade (Attachment 2)

3) Safety variables

・Adverse events
・Laboratory test
・Echocardiography
・Blood pressure and heart rate

4) Criteria for completion of treatment

For subjects in whom the planned treatment period (54-86 weeks) has been completed or who fall under any of the criteria for completion of combined treatment (death, attachment of an invasive respiration assisting device or beginning of all-day respirator use), the dose level is reduced gradually to zero within one month, to complete treatment with the test drug/placebo.

5) Evaluation of safety and efficacy

Statistical analysis of efficacy data at the end of the study is assigned to an outside contractor. The information about the subjects assigned to the active drug treatment and placebo groups is disclosed at the time of statistical analysis. Statistical analysis as to the efficacy of the test drug is carried out in comparison to the efficacy data from the placebo group, the time course of variables relative to estimates at the end of the 12-week observation period and the natural history of the disease.

The ALS Treatment Plan Evaluation Committee evaluates and judges the safety and efficacy of NDDPX08 in patients with ALS on the basis of the results of the statistical analysis.

Interim evaluation is performed when the number of subjects treated with NDDPX08 for 6 months has reached 25. At that time, statistical analysis of efficacy variables is carried out in comparison to the time course of variables relative to the estimates at the end of the 12-week observation period and the natural history of the disease. At the time of interim evaluation, information as to the subjects assigned to the active drug treatment and placebo groups is not disclosed.

9. Evaluations/tests and their schedules

The evaluations and tests specified below are carried out during the study in accordance with the schedules given in Fig. 2.

Safety is evaluated by the attending physician for each subject. Efficacy is evaluated by one or more neurologists not serving as the attending physician.

Fig. 2 Flow chart of the clinical study (evaluation and survey schedule)

		12-week observation period		NDDPX08 treatment period						Upon discontinuation
				Start	After start				Every other month after 14th week	
		Start	6 weeks		2 weeks	4 weeks	8 weeks	14 weeks		
Informed consent, registration		○		○						
Background	Background variables	●								
	Body weight	●	●	●			●	●	●	
	Complications/disease history	●								
	Concomitant drugs/therapy	●	←——	——	——	——	——	——	——→	●
Efficacy	ALSFRS-R	●	●	●		●	●	●	●	●
	%FVC	●	●	●			●	●	●	●
	Modified Norris Scale	●	●	●			●	●	●	●
	ALSAQ-4Q	●	●	●			●	●	●	●
	Manual muscle test (MMT)	●	●	●			●	●	●	●
	Pinching power	●	●	●	●	●	●	●	●	●
	Grip	●	●	●	●	●	●	●	●	●
	ALS severity grade	●	●	●			●	●	●	●
Safety	Laboratory tests	●	●	●		●	●	●	●	●
	Echocardiography	●*1								●
	Blood pressure/heart rate	●	●	●	●	●	●	●	●	●
	Adverse events*2	←——	——	——	——	——	——	——	——→	○ 1 month after last dose

*1 Echocardiography is performed before the observation period.

*2: Presence/absence of adverse events is checked during the period from the start of treatment until one month after the last dose.

1) Background variables

Date of informed consent, gender, birth date, hospitalization status (inpatient/outpatient), diagnosis, body weight, height, complications, disease history, prior treatment, etc. are checked.

2) Evaluations

ALSFRS-R score, %FVC, Modified Norris Scale score, ALSAQ-40 score, MMT (manual muscle test), pinching power, grip and ALS severity grade are rated and entered in the case report form.

3) Laboratory tests

The following laboratory parameters are measured, and the presence/absence of clinically relevant abnormalities and their details are entered in the case report form.

(1) Hematology: RBC, hemoglobin, hematocrit, WBC, platelet count, differential leukocyte count

(2) Biochemistry: AST, ALT, LDH, ALP, γ-GTP, total bilirubin, glucose, total protein, albumin, total cholesterol, BUN, creatinine, CK, uric acid, Na, K, Cl

(3) Urinalysis: protein, glucose, occult blood, urobilinogen

4) Echocardiography

Echocardiography is carried out prior to the clinical study to check for abnormalities of heart valves such as valvular hypertrophy, restricted range of valve motion and stenosis associated with such abnormalities, etc. The echocardiographic findings are entered in the case report form. (Patients with moderate disease involving only one valve are eligible to participate in the study.)

5) Blood pressure and heart rate

Blood pressure and heart rate are measured in the sitting position and their readings are entered into the case report form.

6) Adverse events

Each patient is checked by interview for adverse events during each visit. Particular attention is paid to hallucination, nausea-vomiting and anorexia. Upon the onset of any adverse event, its nature, time of onset and duration, severity, intervention, outcome, causal relationship with the study drug and seriousness are recorded.

10. Predictable adverse events

Of the 5,212 patients with Parkinsonian syndrome previously treated with the test drug, 978 (18.8%) experienced adverse reactions, including gastrointestinal symptoms (nausea/vomiting 433 cases, 8.3%, anorexia 128 cases, 2.5%, gastric discomfort 57 cases, 1.1%, constipation 39 cases, 0.7%, oral thirst 31 cases, 0.6%, and others), neuropsychiatric symptoms (hallucination/delusion 145 cases, 2.8%, dyskinesia 106 cases, 2.0%, dizziness/vertigo 60 cases, 1.2%, headache/dull headache 57 cases, 1.1%, and others) and cardiovascular symptoms (orthostatic syncope 36 cases, 0.7%, and others).

In a previous study of 99 women who required suppression of milk output after labor, abortion or premature labor, 12 of the 53 patients treated with this drug at a dose level of 5 mg/day showed a total of 25 adverse reactions, including nausea (4 cases), vomiting (2 cases), constipation (8 cases), dizziness/vertigo (7 cases), malaise (3 cases) and gastric pain (1 case). No abnormal changes were noted

in any laboratory parameters.

11. Handling of adverse events

1) Symptoms or diseases

All unfavorable or unintended signs, symptoms or diseases arising during the study period are counted as adverse events. Exacerbation of complications present at the start of treatment with NDDPX08 (test drug) is also counted as an adverse event. Worsening of an efficacy variable is not counted as an adverse event.

2) Objective findings

An abnormal change in any laboratory parameter from its value at the start of the study* (normal→ abnormal or abnormal→more abnormal) is counted as an adverse event. If a laboratory parameter with a missing value at the start of the study* is abnormal after the start of treatment, it is counted as an adverse event. In cases where the value of a laboratory parameter at the start of the study is missing, the value recorded within 30 days before informed consent is used as a reference value.

*· The value recorded during the observation period after informed consent (the value recorded at a time point closer to the start of treatment, if there were two or more measurements during the observation period).

For each adverse event, regardless of whether or not there is some description of such an event in the protocol, the date of onset, the time of maximum exacerbation, the time of outcome judgment and data needed for judgment of a causal relationship with the study drug are entered in the case report form.

3) Recording and investigation of adverse events

Upon the onset of an adverse event, entry in the column "Adverse events" of the case report form is needed as to the symptom/condition, objective findings, date of onset, severity, seriousness, presence/absence of intervention and its contents, outcome, date of outcome judgment, causal relationship to the study drug and the reason for such a causality judgment. When the name of the disease arising as an adverse event is entered in this column, the symptoms accompanying the disease do not need to be entered as adverse events.

If a symptom, disease or objective finding is observed or obtained as an adverse event during the study, it is followed until it subsides to a level viewed as normal or no longer viewed as an adverse event, as a rule, regardless of the presence/absence of a causal relationship with the study drug (see "Criteria for handling of test findings", as regards objective findings). Such follow-up is not needed if the Investigator or Sub-Investigator judges the patient to have recovered from the adverse event(s). In the latter case, the rationale for the judgment of recovery is entered in the case report form. For each irreversible adverse event involving organic disorders (cerebral infarction, myocardial infarction, etc.), follow-up is continued until stabilization or fixation of the symptom.

4) Classification of adverse events

The severity of adverse events is rated on the scale given below.

(a) Mild: No disturbance in the daily life of the patient

(b) Moderate: Some disturbances in the daily life of the patient, but activities of daily life possible if the patient endures considerable inconveniences

(c) Severe: Serious disturbances in the daily life of the patient

The outcomes of adverse events are rated on the scale given below.

(1) Recovery: The event has subsided to a level viewed as normal or no longer viewed as an adverse event

(2) Persistence: Recovery not seen at a given time point

(3) Unknown (dead): Outcome unknown because the patient is dead

5) Association between adverse events and the study drug

The association of adverse events with the study drug is rated on the scale given below, taking into account the subject's condition, temporal relationship to drug treatment, the possibility of involvement of other factors and so on.

(1) Definitely present

(2) Probably present

(3) Possibly present

(4) Not related

Of the adverse events, those rated as category (1) to (3) are counted as adverse events whose causal relationship to the study drug cannot be ruled out. Adverse events rated as category (4) are counted as adverse events whose causal relationships to the study drug are ruled out.

6) Serious adverse events

Upon the onset of a serious adverse event during the study, regardless of the presence/absence of a causal relationship to the study drug, the Investigator or Sub-Investigator is required to immediately take appropriate actions for the subject concerned. In addition, the Investigator needs to immediately report the event to the head of the participating facility.

【Serious adverse events】

(1) Death

(2) Events possibly leading to death

(3) Events requiring hospitalization or extension of hospitalized period

(4) Events causing disabilities

(5) Events possibly leading to disabilities

(6) Events of a serious nature, resembling those listed above in (1) through (5)

(7) Congenital illness or abnormalities in the next generation

7) Supply of new information

Upon acquisition of new information about the safety of this study, the Investigator is required to immediately report such information in writing to the head of the participating facility and the Sub-Investigator. The Investigator or Sub-Investigator is required to provide additional explanation(s) to the subjects and must revise the leaflet and informed consent form, if needed.

12. **Payment of expenses and compensation**

1) Treatment expenses

Routine doctor fees, drug costs and transportation costs related to the study are borne by each subject.

However, the costs of NDDPX08 and tests related to this study (specified in Fig. 2, Page 18) are paid by the Ministry of Health, Labour and Welfare Scientific Research Program.

2) Compensation

Upon the onset of any health hazard to the subjects arising from this study and requiring compensation or liability for damages, how to deal with it is decided through discussions among the Director of Tokai University School of Medicine, the Director of the Tokai University Hospital, the Study Director and the Investigator.

13. Statistical analysis (Efficacy evaluation)

1. Population analyzed

(1) Case handling

The representative of the researchers (Representative Researcher) decides on the handling of the data from individual subjects and fixes the case at the end of the study in accordance with the rules set forth in this paragraph. In cases where the rules on case handling set forth in this paragraph are not applicable, the Representative Researcher prepares case handling rules for such individuals and decides on the handling of their cases in accordance with the procedure stipulated in the "Ethical Guidelines on Clinical Studies" (or Standard Operating Procedure on GCP). If the Representative Researcher has prepared new rules on case handling, the rules need to be entered into the statistical analysis schedule which is finally fixed before opening of the information regarding active drug/placebo allocation.

1) Full Analysis Set (FAS)

FAS is composed of all subjects except those listed below.

Cases with disease not covered by this study
· Cases seriously violating the GCP
· Cases not treated with the test drug/placebo
· Cases where no data on efficacy are available

2) Per Protocol Set (PPS)

PPS is composed of FAS members except the subjects listed below.

· Cases not satisfying the inclusion criteria
· Cases falling under any of the exclusion criteria
· Cases where any of the prohibited concomitant drugs began to be used after the start of the study

3) Safety analysis set

Safety analysis set is composed of all subjects except those listed below.

· Cases seriously violating the protocol (and GCP)
Cases not treated with the test drug/placebo
Cases where no data on safety are available

(2) Data handling

1) Date of observation, tests and investigation

Data from observations, tests and investigations collected during the below-shown period (acceptable range of dates) are adopted for analysis. Data from observations, tests and investigations not collected within this period are not adopted for analysis and are counted as missing data.

2) Efficacy data

Lack of measurement of any of the following: ALSFRS-R, %FVC, Modified Norris Scale, ALSQ40, grip, pinching power and ALS severity grade. These are all counted as missing data.

The acceptable range of dates for each evaluation and measurement is specified below.

【Occasion】	【Acceptable range of dates】
Temporary registration:	Not set
Before formal registration:	77 days (11 weeks) – 91 days (13 weeks) after ALSFRS-R evaluation before temporary registration, and 7 days before the start of treatment – immediately before the start of treatment
After the start of treatment:	The planned date of evaluation and three days before and after the planned date of evaluation
Upon discontinuation (ceased drug treatment):	Date of discontinuation – 7 days after discontinuation

3) Safety data

If a laboratory or sensory test has not been performed or is impossible to perform due to problems related to samples, etc. or if the data from the test can be used only as reference data, the data concerned are counted as missing data.

The acceptable range of dates for each evaluation and measurement is specified below.

【Occasion】	【Acceptable range of date】
Before temporary registration:	Informed consent – Immediately before temporary registration
Before formal registration:	7 days (11 weeks) before formal registration – immediately before formal registration (test data need to be available by the time of formal registration)
Before the start of treatment:	7 days before the start of treatment – Immediately before the start of treatment
After the start of treatment:	The planned date of evaluation and three days before and after the planned date of evaluation
Upon discontinuation (ceased drug treatment):	Date of discontinuation – 7 days after discontinuation

If additional rules on data handling are needed, the Representative Director decides such rules in accordance with the procedure stipulated in the "Ethical Guidelines on Clinical Studies" (or Standard Operating Procedure on GCP) and enters them into the statistical analysis schedule which is finally fixed before opening of the key code for the test drug/placebo allocation.

2. Statistical analysis schedule

The protocol sets forth the methods of statistical analysis only to the minimal extent needed to achieve the primary goals of the study. Details and technical aspects of the statistical analysis methods are specified in the statistical analysis schedule which is prepared separately by the start of treatment for the

first case. The statistical analysis schedule is fixed before opening of the key code for the test drug/placebo allocation.

(1) Analysis of demographic and other characteristics

Inter-group uniformity of the following demography and other variables is analyzed by Fisher's exact text, chi-square test or two-sample t-test selected depending on the nature of the data scale. Significance level is set at 15% (two-tailed).

【Variables analyzed】

Gender, age, body weight, height, presence/absence of complications

ALS type (solitary/familial; EL Escorial Modified Airlie House Diagnostic Criteria)

Duration of sickness, initial symptoms, ALS severity grade

Presence/absence of concomitant drugs, presence/absence of concomitant therapy

On variables found to lack inter-group uniformity and selected based on medical considerations, the presence/absence of interactions is analyzed and the analysis adjusted for these variables is carried out.

(2) Efficacy variables

Efficacy variables are analyzed in FAS. To confirm the stability of this analysis, PPS is additionally analyzed as needed.

1) Primary efficacy variable

As to the ALSFRS-R score (the primary efficacy variable), summary statistics (mean, standard deviation, minimum, median and maximum) are calculated for each group and time point, as well as summary statistics of the difference between the pre-treatment score (during the observation period) and the score at each point after the start of treatment.

(a) Primary analysis

Analyses (i) and (ii), shown below, are carried out. If a significant difference between the NDDPX08 and placebo groups is revealed by any of the analyses, the efficacy of NDDPX08 is judged to have been verified. The 95% confidence interval for the inter-group difference is calculated as reference information for interpretation of the study results.

(i) The difference between "the score before the start of treatment after the observation period" and "the score at 2 weeks after the end of 15 mg/day treatment (after completion of the dose escalation period) or upon discontinuation of the treatment" is subjected to analysis of covariance and inter-group comparison, with the factors used for dynamic allocation (severity and age) serving as co-variables. In cases where the data at 2 weeks after the end of treatment are missing, data are derived by the LOCF (Last Observation Carried Forward) method.

(ii) The score at each time point after the start of treatment relative to the score "before the start of treatment after the observation period" is subjected to repeated measures analysis of covariance and inter-group comparison, with the factors used for dynamic allocation serving as co-variables (analysis for each treatment group, each time point and interactions between treatment group and time point).

(b) Secondary analyses

For each subject, the mean of the difference between the score "before the start of treatment after the observation period" and each time point after the start of treatment is calculated, to yield summary statistics. This is followed by analysis of covariance and inter-group comparison, with the factors used for dynamic allocation serving as co-variables.

Simple regression analysis is carried out for each subject, and the gradient of the line representing the changes over time is calculated to yield summary statistics. This is followed by analysis of covariance and inter-group comparison, with the factors used for dynamic allocation serving as co-variables.

Analyses with the time course taken into account, using statistical models (e.g., analysis with mixed efficacy model, etc.), are carried out.

· Survival time analysis using the events defined by the ALSFRS-R score is carried out.

2) Secondary efficacy variables

To determine the time of death or disease progression to a certain state, survival time analysis is carried out. For this analysis, defined events are death, loss of unassisted gait capability, loss of arm function, loss of leg function, tracheotomy, attachment of a respirator and tube feeding, and discontinuation is defined as cessation of treatment for other reasons. Analysis is conducted on %FVC, Modified Norris Scale score, ALSAQ40 score, grip and pinching power, using a method similar to that employed for analysis of the ALSFRS-R score. A shift table is prepared, representing the time course of ALS severity grade for each group from "before the start of treatment" until "2 weeks after the end of 15 mg/day treatment (after completion of the dose escalation period) or upon discontinuation of the treatment."

(3) Safety variables

Safety is analyzed in the safety analysis set. For each group, the incidences of adverse events, adverse reactions, serious adverse events and serious adverse reactions are calculated, followed by inter-group comparison using Fisher's exact test.

On each parameter of laboratory tests and one sensory test (vibration sense), the summary statistics at each time point after the start of treatment and their difference from their measured values before the start of treatment are calculated for each group. In addition, the percentage of subjects showing abnormal changes in any parameter is calculated for each group.

A shift table is prepared, representing the time course of each parameter of urinalysis and sensory tests (numbness and vertigo) at each time point after the start of treatment relative to the measured values before the start of treatment for each group.

(4) Significance level

The significance level is set at 5% (two-tailed). The confidence interval (bilateral) is calculated with a confidence coefficient of 95%. In view of the special circumstances at the time of planning of this study, adjustment of the significance level for a multiplicity of tests in the primary analysis is skipped.

14. Publication of results

The privacy of each subject is preserved when the results of this study are made public.

15. References

1) Tanaka K, et al.. A dopamine receptor antagonist L-745, 870 suppresses microglia activation in spinal cord and mitigates the progression in ALS model mice. Exp Neurol. 2008 Jun; 211(2):378-86. Epub 2008 Mar 4.

2) Okada Y, et al.. A dopamine D4 receptor antagonist attenuates ischemia-induced neuronal cell damage

via upregulation of neuronal apoptosis inhibitory protein. J Cereb Blood Flow Metab. 2005 Jul; 25(7):794-806.

3) Societas Neurologica Japonica: Guidelines on Treatment of ALS 2002. Clinical Neurology 42(7), 678-719

4) Ohashi Y, et al.: Use of the ALS Functional Rating Scale (Revised Japanese Version) for evaluation of activities of daily living by patients with amyotrophic lateral sclerosis (ALS). Noshinkei 53(4)346-355, 2001

5) Yamaguchi T, et al.: ALS-specific QOL Scale ALSAQ-40 (Japanese Version) – Its validity and clinical application. Noshinkei 56(6)483-494, 2004

6) Societas Neurologica Japonica: Precautions on the Use of Dopamine Agonists (May 22, 2008)
