# Supplementary material for: Correction: Bromocriptine Mesylate Attenuates Amyotrophic Lateral Sclerosis: A Phase 2a, Randomized, Double-Blind, Placebo-Controlled Research in Japanese Patients
Source: PLoS One. 2016 Mar 28;11(3):e0152845. doi: 10.1371/journal.pone.0152845 (PMC4809614; doi:10.1371/journal.pone.0152845)
Supplement: S1 File — (PDF) [file pone.0152845.s001.PDF]

PLOS ONE

RESEARCH ARTICLE

Bromocriptine Mesylate Attenuates Amyotrophic Lateral Sclerosis: A Phase 2a, Randomized, Double-Blind, Placebo-Controlled Research in Japanese Patients

Eiichiro Nagata[1], Mieko Ogino[2], Kounosuke Iwamoto[3], Yasuhisa Kitagawa[4], Yasuo Iwasaki[3], Fumihito Yoshii[5], Joh-E. Ikeda[6,7]*, ALS Consortium Investigators¶

1 Department of Neurology, Tokai University School of Medicine, Isehara, Japan, **2** Department of Neurology, Kitasato University School of Medicine, Sagamihara, Japan, **3** Department of Neurology, Toho University Omori Medical Center, Tokyo, Japan, **4** Department of Neurology, Tokai University Hachioji Hospital, Tokyo, Japan, **5** Department of Neurology, Tokai University Oiso Hospital, Kanagawa, Japan, **6** Molecular Neurology, Faculty of Medicine, Kitasato University School of Medicine, Sagamihara, Japan, **7** Department of Pediatrics, Faculty of Medicine, University of Ottawa, ARC/Children's Hospital of Eastern Ontario, Ottawa, Canada

¶ Membership of the ALS Consortium Investigators is listed in the Acknowledgments.
* joh-e@mgcheo.med.uottawa.ca

OPEN ACCESS

Citation: Nagata E, Ogino M, Iwamoto K, Kitagawa Y, Iwasaki Y, Yoshii F, et al. (2016) Bromocriptine Mesylate Attenuates Amyotrophic Lateral Sclerosis: A Phase 2a, Randomized, Double-Blind, Placebo-Controlled Research in Japanese Patients. PLoS ONE 11(2): e0149509. doi:10.1371/journal.pone.0149509

Editor: Yun Wang, National Health Research Institutes, TAIWAN

Received: July 9, 2015

Accepted: February 1, 2016

Published: February 24, 2016

Copyright: © 2016 Nagata et al. This is an open access article distributed under the terms of the Creative Commons Attribution License, which permits unrestricted use, distribution, and reproduction in any medium, provided the original author and source are credited.

Data Availability Statement: Data are available from UMIN website (https://upload.umin.ac.jp/cgi-open-bin/ctr/ctr.cgi?function=brows&action=brows&type=summary&recptno=R000005640&language=E).

Funding: This work was supported by Ministry of Health, Labor and Welfare, Japan (H20-ClinicalResearch-G-014).

Competing Interests: The authors have declared that no competing interests exist.

Abstract

Objective

Bromocriptine mesylate (BRC), a dopamine D2 receptor agonist has been shown to confer neuroprotection, sustained motor function and slowed disease progression in mouse models of amyotrophic lateral sclerosis (ALS) Here we report a first in human trial in ALS.

Design

A multicenter, Riluzole add-on, randomized, double-blind, placebo controlled 102-week extension BRC clinical trial.

Methods

The trial was conducted between January 2009 and March 2012 on 36 Japanese ALS patients. A 12-week treatment with Riluzole observational period was followed by combined treatment (Riluzole + BRC; n = 29 or Riluzole + placebo; n = 7). The dosing commenced at 1.25 mg/day increasing in steps at two weeks intervals to a maximum of 15 mg/day. The efficacy of BRC was evaluated by comparing BRC and placebo groups upon completion of stepwise dosing at 14 weeks 2 points (1st endpoint) and upon completion or discontinuation of the study (2nd endpoint) of the dosing.

Results

Statistics analyses revealed a marginal BRC treatment efficacy with P≦20%to placebo by 1st and 2nd endpoint analysis. In the 1st endpoint analysis, BRC group was significantly

effective on the scores of ALSAQ40-communicaton (P = 1.2%), eating and drinking (P = 2.2%), ALSFRS-R total (P = 17.6%), grip strength (P = 19.8%) compared to the placebo group. In the 2nd endpoint analysis, differences between the scores of Limb Norris Scale (P = 18.3%), ALSAQ40-communication (P = 11.9%), eating and drinking (P = 13.6%), and neck forward-bent test (P = 15.4%) of BRC group were detected between the two groups. There was no significant difference between the treatment groups for adverse events or serious drug reactions incidence.

Conclusions

BRC sustains motoneuronal function at least in part through BRC treatment. Further analysis involving a Phase 2b or 3 clinical trial is required but BRC currently shows promise for ALS treatment.

Trial Registration

UMIN Clinical Trials UMIN000008527

Introduction

Amyotrophic lateral sclerosis (ALS) is a neurodegenerative disorder that preferentially targets motor neurons controlling muscle movement and is usually fatal. Approximatley 90% of ALS cases occur sporadically in the absence of a clear family history, the remainder have a clear genetic background and are diagnosed as familial ALS [1,2]. While the precise etiology or etiologies of ALS remains unknown, a complex interplay of many pathogenic factors, including oxidative stress, excitotoxicity, mitochondrial dysfunction, disruption of neurofilament network, neural inflammation, non-cell autonomous damage and protein aggregations such as mutant SOD1, TDP-43, and FUS proteins, have been suggested as possible potential factors [3–6]. Notably, an elevation of reactive oxygen species (ROS) possibly reflecting mitochondrial dysfunction [3,6], the reduction of glutamate uptake in motor neurons, and induction of oxidation in neighboring astrocytes [7] point to roles for oxidative stress and inflammatory response in ALS [8]. We believe that oxidative stress therefore represents a credible target for the development of novel therapeutic agents for ALS. We have previously developed the neuronal apoptosis inhibitory protein (NAIP)-ELISA-based drug screening system [9]. NAIP is a founding member of anti-apoptosis IAP family [10] and selectively suppresses oxidative stress-induced cell death. Both upregulation and exogenous over expression of NAIP protects neuronal cells against oxidative stress in vivo and in vitro [11–13]. Using this system, we have identified several compounds that transiently upregulate NAIP, including bromocriptine methylate (BRC) [9,13]. BRC confered protection against oxidative stress-induced cell death [14,15]. Currently the only Food and Drug Administration (FDA)-approved drug for the treatment of ALS, the anti-glutamatergic Riluzole, confers only a modest therapeutic efficacy [16,17]. Therefore, there is a clear unmet need for effective ALS therapeutic interventions [18]. The present study is designed to evaluate both the efficacy of BRC in the presence of Riluzole on motor function and quality of life (QOL) as well as safety in Japanese patients with solitary ALS.

Materials and Methods

Participants and study design

A double blind, Riluzole add-on placebo controlled BRC safety and efficacy trial in Japanese ALS patients was designed. The study protocol (08R-103) was approved by the Institutional Review Board for Clinical Research, Tokai University, followed the tenets of the Declaration of Helsinki and is registered at UMIN (UMIN000008527). The approved protocol for this trial and supporting CONSORT checklist are available as supporting information; see S1 Protocol and S1 CONSORT Checklist. All participants provided written informed consent. This is a Phase 2a clinical trial to evaluate the safety and the efficacy of BRC. We determined 50 ALS patients who lived around Tokyo metropolitan area suburbs in Japan.

48 ALS patients were recruited from 3 university hospitals and 4 general hospitals in Japan between January 1st, 2009 and March 31th, 2012. We enrolled patients with a diagnosis of possible, laboratory-supported probable, probable, or definite ALS in accordance with the revised El Escorial Modified Airlie House Diagnostic criteria [19]. ALS was diagnosed in all study entrants within 3 years of the inception of our study. The forced vital capacity (FVC) of patients was more than 70%. The ages of patients were between 20 and 75 at the time of taking informed consent. The changes in ALSFRS-R scores of patients were between -1 and -4 during the pretreatment 12-week observation period. All study entrants were treated with 100mg of Riluszole (50mg a twice daily) during the course of this study. 10 participants were disqualified during 12 weeks observation period (Fig 1). The final number of participants was 38 (BRC group: n = 31, control (placebo group: n = 7)). 10 participants were withdrawn during the course of study. Eight participants withdrew from the BRC group following adverse events such as nausea, constipation, there was one death in the placebo group, and one participant in the BRC group left the study of his own accord. In the placebo group, one participant died secondary to ALS progression. In the full analysis set (FAS) to efficacy assessment, 2 patients were disqualified (BRC n = 2, placebo n = 0). In one patient, his secrecy obligation was not defended. In other patient, we could not get the validity at all about this medicine. In per protocol set (PPS) to efficacy assessment, there were no unqualified patients. No significant differences between BRC and placebo groups was detected for the baseline characteristics including age, gender, body weight, BMI, ALS duration, family history, ALSFRS-R score, and vital capacity (Table 1).

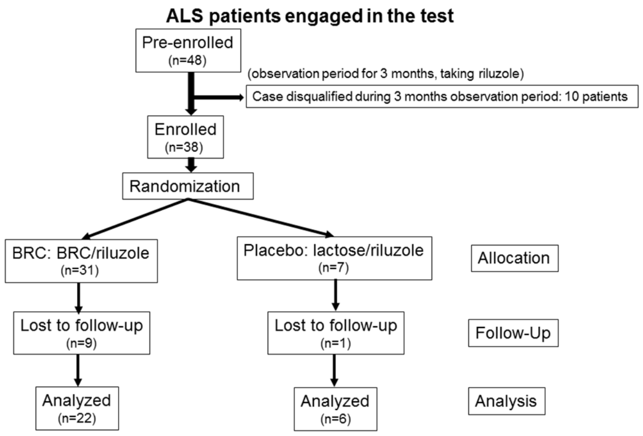

Fig 1. Trial profile. BRC: bromocriptine methylate.

doi:10.1371/journal.pone.0149509.g001

Table 1. Summary of demographic and baseline characteristics for randomized groups in BRC and placebo.

	BRC (n = 29)	Placebo (n = 7)
Age/ years	59.7(±9.0)	58.7(±10.1)
Male	21(72.4%)	6(85.7%)
Female	8(27.6%)	1(14.3%)
Body weight/ Kg	58.08(±9.47)	58.97(±7.53)
Body-mass index/ kg/m2	21.9	21.6
Duration of symptoms/ years	1.45(±0.69)	1.60(±0.77)
Family history of amyotrophic lateral sclerosis	NA	NA
Baseline ALSFRS-R score	40.0(±5.0)	39.9(±5.6)
Mean vital capacity at baseline	105.03(±20.30)	92.19(±14.89)
ALS severity/ patients		
I	8(27.6%)	1(14.3%)
II	11(37.9%)	3(42.9%)
III	10(34.5%)	3(42.9%)
unknown	0(0.0%)	0(0.0%)

We classified clinical manners of ALS severity of five grades established by Japanese Ministry of Health, labor and Welfare as follows.

ALS severity

I: Housekeeping and working are possible in general.

II: Although housekeeping and working are impossible, daily life becomes independent in general.

III: Meal, excretion, and any one or more movements, but daily life takes care.

IV: Breathing difficulty, sputum excretion difficulty, or swallowing difficulty.

doi:10.1371/journal.pone.0149509.t001

Randomisation and masking

This investigator initiated study was designed as a Riluzole add-on, randomized, double-blind, placebo-controlled trial. Efficacy and safety were evaluated through analysis of change in each variable measured at the beginning of the observation period and at the commencement and the end of the treatment with BRC/ Riluzole/ lactose (BRC arm) and Riluzole/ lactose (placebo arm). The participants were blinded and we randomly allocated them in a 4: 1 ratio to receive each drugs using a computer-generated random allocation sequence.

Procedures

For patients who had been dosed Riluzole 4 weeks prior or on time of the observation period, the 12-week treatment with 100mg Riluzole alone (50mg twice daily) during the observation period was performed and followed by the either BRC or placebo treatments. The initial dose of 1.25 mg BRC per day was increased to 15 mg/day at 2-week intervals over 12-weeks (Fig 2). The dosing period from 58 to 90 week ended in a 4 week stepwise dose reduction followed by a one month observational period following the completion of dosing. When a dose-related adverse effect was observed rendering the, for example, 10mg/day difficult to maintain, a reduced dose of 7.5 mg/day was used as the maintenance dose. One patient took 10mg/day of BRC as the maintenance dose for his nausea.

Statistical analyses of ALSFRS-R, %FVC, modified Norris scale (limb and bulbar), ALSAQ-40, manual muscle testing (MMT), pinch strength and grip strength were performed.

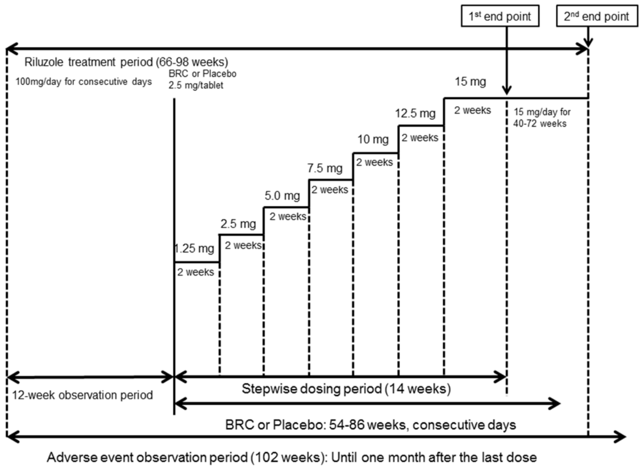

Fig 2. Flow chart of the clinical study (drug treatment method): BRC stepwise dosing schedule. There was no significant difference on each heading between BRC and Placebo groups.

doi:10.1371/journal.pone.0149509.g002

Outcomes

The BRC and placebo identities were revealed once the dosing was completed and we evaluated efficacy by two statistical analyses. The 1st endpoint analysis compared baseline scores at the end of the observation period to those upon completion of stepwise dosing of 14 weeks (Fig 2). The 2nd endpoint analysis compared the baseline score at the observation period endpoint to that at the time of study completion (Fig 2).

Primary efficacy analysis I (ANCOVA)

Efficacy was estimated by a comparison of BRC and placebo groups mean scores at 1st and 2nd endpoints. Analysis of covariance (ANCOVA) was applied ($p<0.05$).

Primary efficacy analysis II

The primary efficacy analysis II was a comparison of the mean of time-course score changes in BRC and placebo groups.

Efficacy and marginal significance were derived from a p-value of either primary efficacy analysis I (ANCOVA) or primary efficacy analysis II. Marginal significance was defined as a P-value within two-tailed test at 80% confidence level. Moreover, the time-course of the values of summary statistics, mean changes from baseline, and the slope of changes were also used in the analysis.

Laboratory data

WBC count with fractionation, liver, and renal function tests as well as blood sugar were measured at regular intervals; electrocardiograms, echocardiograms, chest X-ray and vital signs were also taken.

Statistical analysis

The study was sufficiently powered to independently assess a potential benefit of BRC compared with placebo for ALSFRS-R total scores and survival. Failure analyses undertaken

for ALSFRS-R used the change from baseline to 1st and 2nd endpoints with the last observation carried forward (LOCF) for subjects who discontinued in Part I and Part II. We believe that the LOCF method was appropriate for exploratory analyses because of the very small number of drop-outs and the absence of study entrant deaths. The statistical analysis of this study was conducted by CMIC (CRO/CMO company, Statistical Analysis Division, Tokyo, Japan).

Results

Primary efficacy analysis I (ANCOVA)

Efficacy was analyzed by a comparison of the mean changes calculated at 1st and 2nd endpoints between BRC and placebo groups. Observed differences with P values less than 0.2 at the 1st endpoint included ALSAQ40-communication (P = 0.012, Fig 3(A)), ALSAQ40 eating & drinking (P = 0.022, Fig 4(A)), ALSFRS-R total score (P = 0.176, Fig 5(A)), and grip strength (right hand)(P = 0.198, Fig 6(A)). At the 2nd endpoint analysis, significant differences were observed for Limb Norris Scale (P = 0.183, Fig 7(B)), ALSAQ40-communication

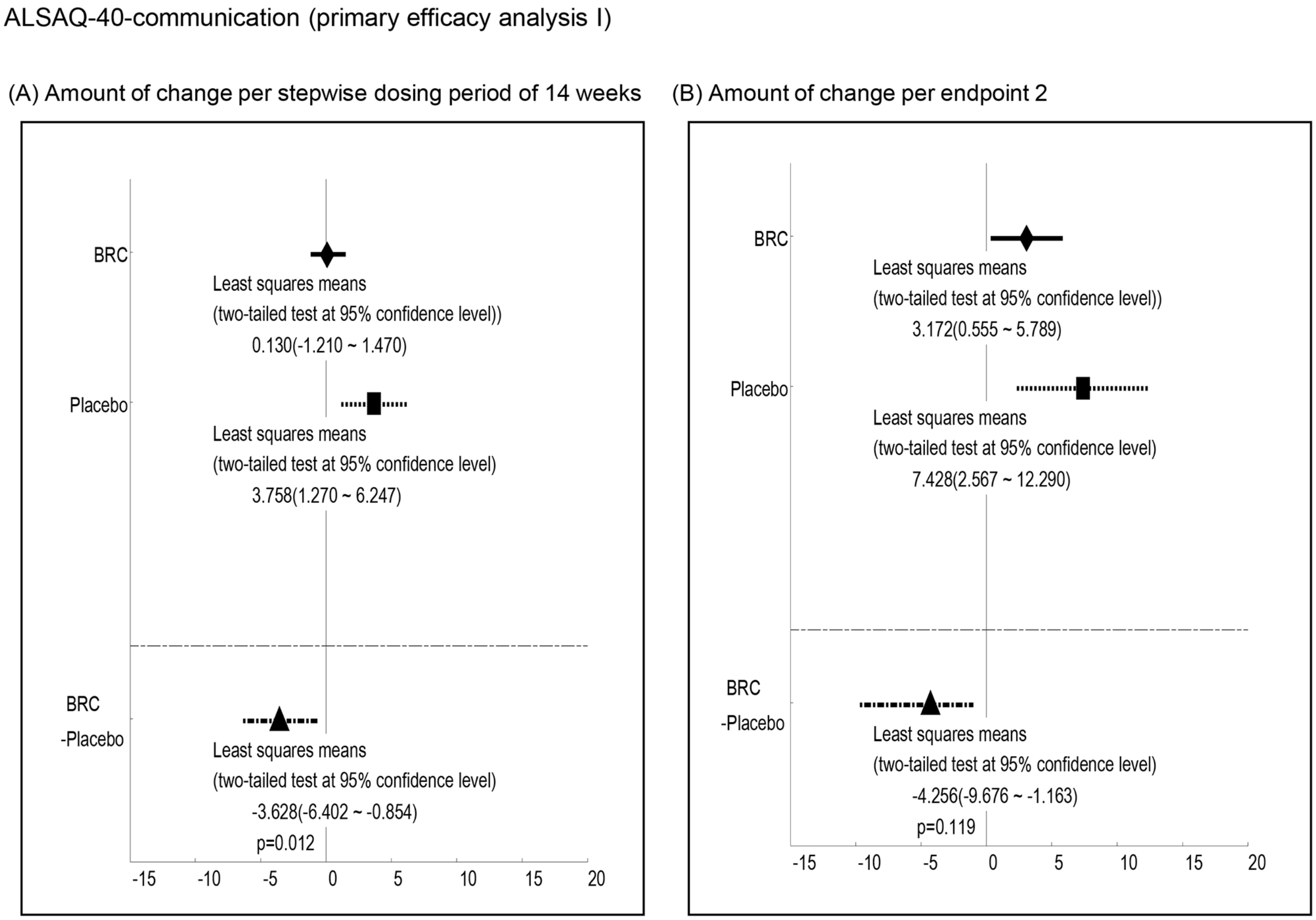

Fig 3. The results of primary efficacy analysis I on Limb Norris Scale total score. In 1st endpoint analysis, the group of BRC treatment tended to be recovered compared to the group of placebo treatment. Moreover, in 2nd endpoint analysis, the group of BRC was significantly recovered in Limb Norris Scale total score compared to the group of placebo (p < 0.2).

doi:10.1371/journal.pone.0149509.g003

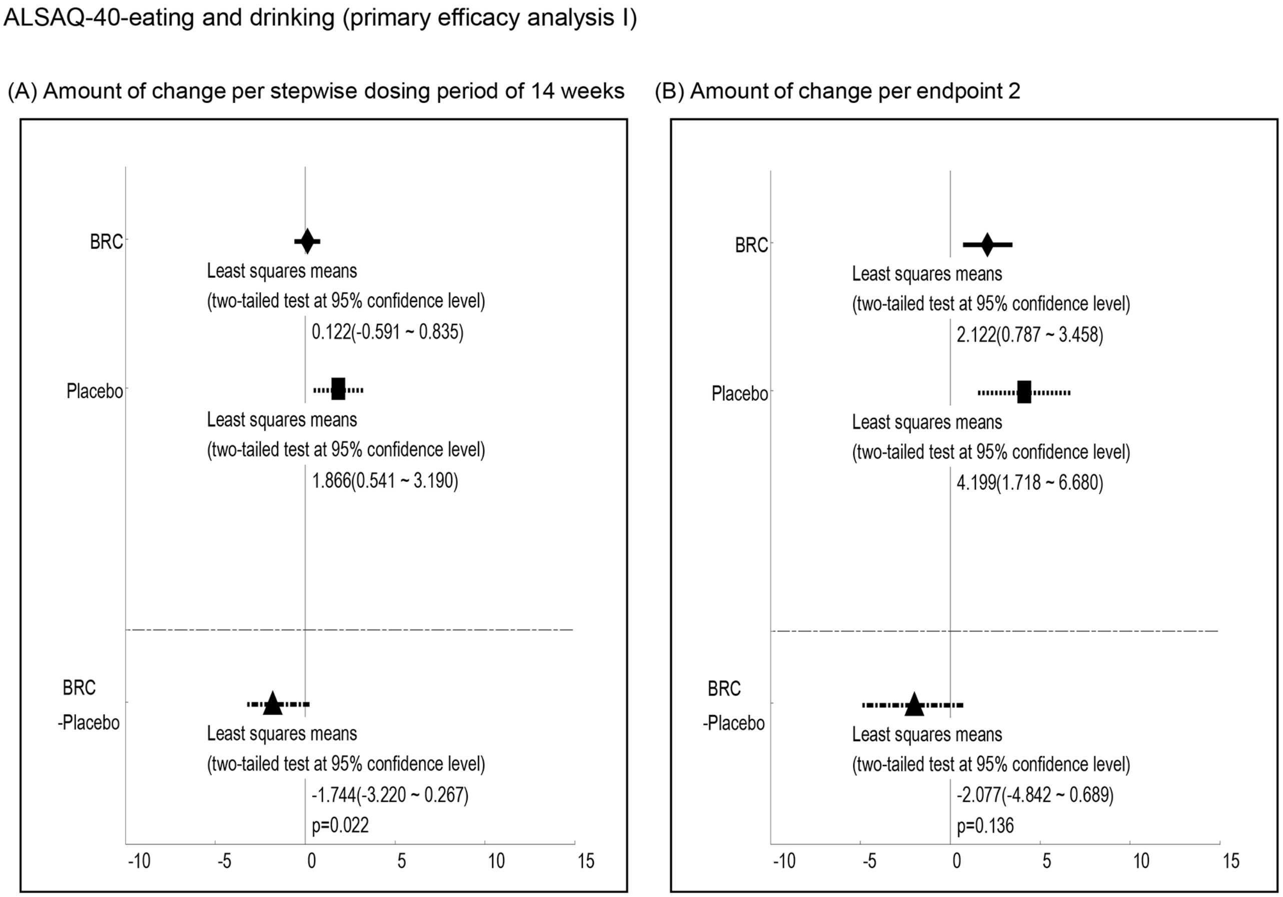

Fig 4. The results of primary efficacy analysis I on ALSAQ40 communication. In both 1st endpoint and 2nd endpoint analyses, the groups of BRC treatment were significantly recovered in ALSAQ40 communication compared to the groups of placebo (p < 0.2).

doi:10.1371/journal.pone.0149509.g004

(P = 0.119, Fig 3(B)), ALSAQ40 eating & drinking (P = 0.136, Fig 4(B)), and degree of neck flexion (P = 0.154, Fig 8(B)).

Primary efficacy analysis II for the comparison of the mean of time-course score changes in BRC and placebo. P values of less than 0.2 were observed for the following items; Limb Norris Scale, ALSAQ40-communication, ALSAQ40 eating & drinking, and grip strength (right hand). Moreover, the abolition rate of lower limb function in ALS treated with BRC was less than that for non-treated ALS patients (Fig 9).

The above ALS scores indicated ALS severity was improved in BRC group compared to Placebo group.

On the other hand, almost patients were diagnosed with "definite" according to ALS diagnosis criteria. Moreover, there was no significant correlation between the efficacy for BRC and ALS progression.

Safety (Table 2)

1. Adverse events

The sole significant differences between the treatment groups were for comparatively minor

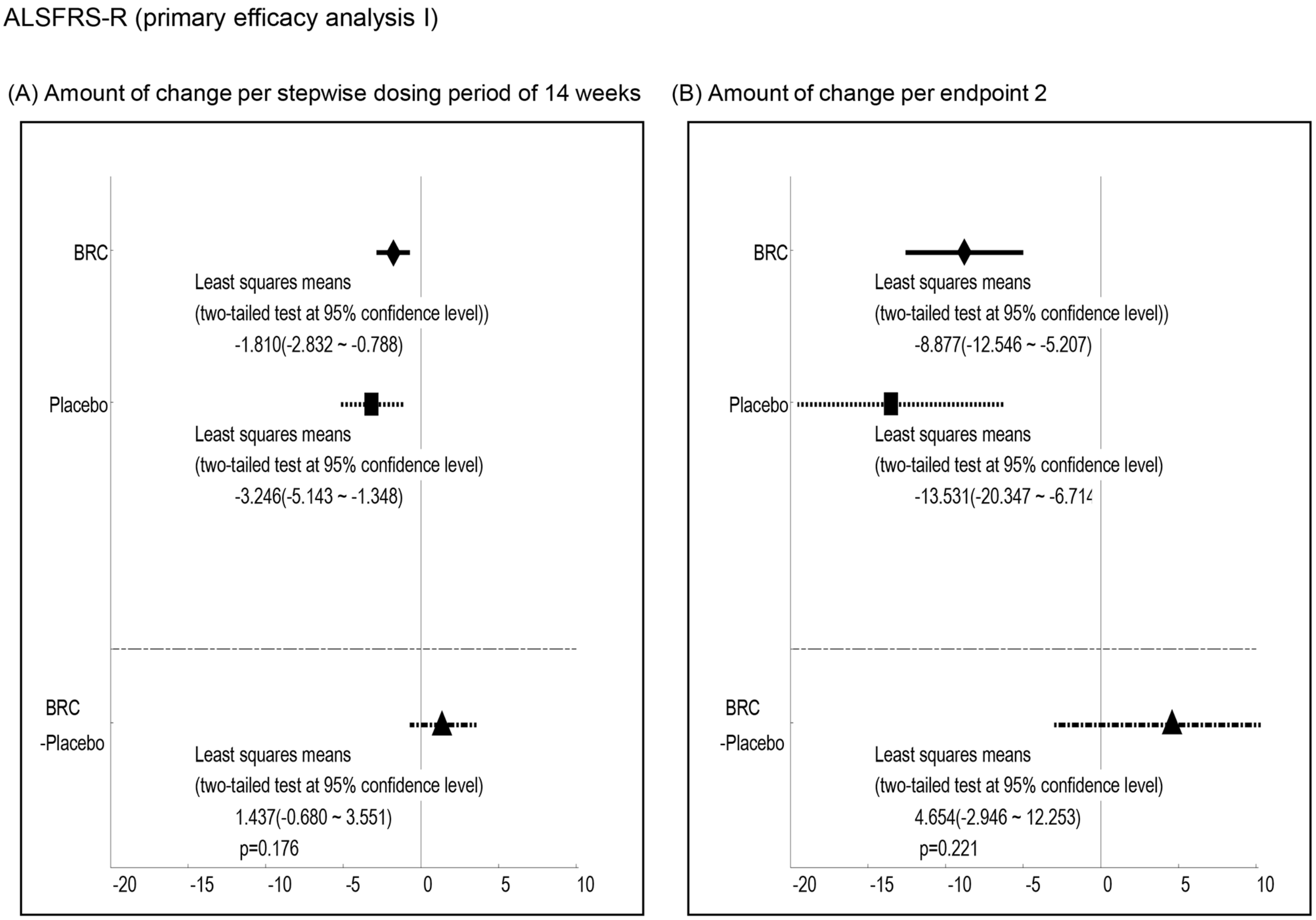

Fig 5. The results of primary efficacy analysis I on ALSAQ40 eating and drinking. In both 1st endpoint and 2nd endpoint analyses, the groups of NDDPX08 treatment were significantly recovered in ALSAQ40 eating and drinking compared to the groups of placebo (p < 0.2).

doi:10.1371/journal.pone.0149509.g005

issues including 19 infections, 14 gastrointestinal disorders, 12 skin and subcutaneous tissue disorders, 11 cases of nasopharyngitis, 5 individuals with constipations, 5 with nausea, and 4 with eczema in BRC group.

2. Adverse drug reactions
   There was no significant difference in incidence between the treatment groups expect for 6 gastrointestinal disorders and 3 nauseas in BRC group.

3. Serious adverse events
   There was no significant difference in serious adverse event incidence between the treatment groups. Three respiratory, thoracic and mediastinal disorders and 2 aspiration pneumonias according to the worse of ALS symptoms in both the treatment groups.

4. Serious adverse drug reactions
   There was no significant difference in serious adverse drug reactions incidence between the treatment groups; there was one case of gastrointestinal disorder and ischemic colitis.

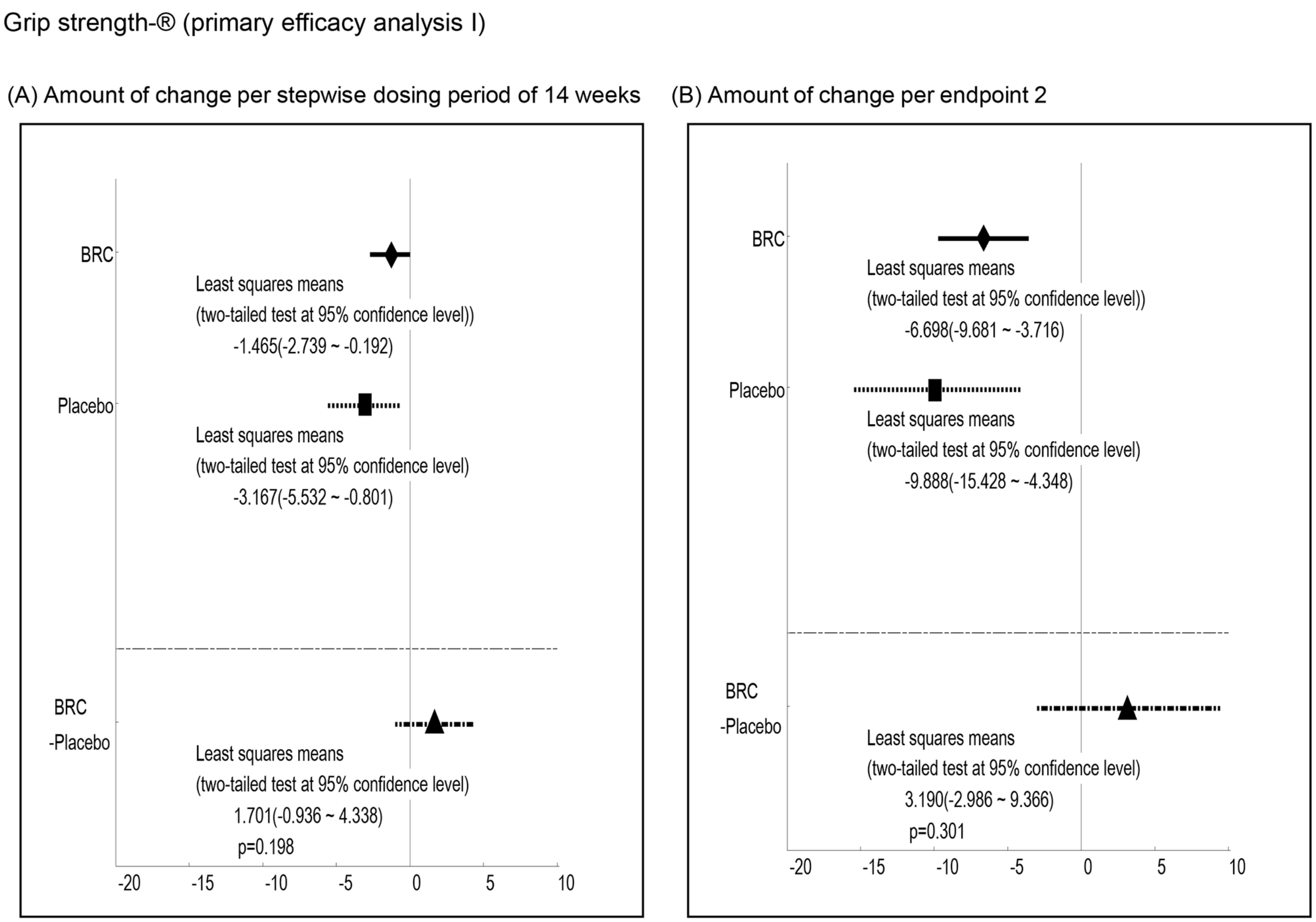

Fig 6. The results of primary efficacy analysis I on ALSFR-S. In 1st endpoint analysis, the group of BRC treatment was significantly recovered compared to the group of placebo treatment ($p < 0.2$). Moreover, in 2nd endpoint analysis, the group of BRC tended to be recovered in ALSFR-S total score compared to the group of placebo.

doi:10.1371/journal.pone.0149509.g006

Laboratory data

All laboratory data were within normal range with the exception of the increasing eosinophils exclusively seen in placebo treated ALS patients (Fig 10).

Discussion

There are a limited number of therapeutic strategies that effectively relieve symptoms and improve the quality of life for ALS patients. Preclinical animal studies are an essential step on the path novel and effective therapeutic agents for the ALS. However, the outcomes of drug efficacy tests using animal models vary due to different methodological conditions such as the differences in giving pre-symptomatic versus post-symptomatic administration of agent [20]. In this regard, standard operating procedures for the preclinical animal study for ALS/MND has recently been published and strongly recommends the exclusive post-onset administration of candidate agents to approximate the clinical reality [21]. Previous studies have shown that dexpramipexole, a dopamine D2 receptor agonists used for Parkinson disease therapy might have the efficacy as an ALS treatment. Dexpramipexole is thought to enhance mitochondrial

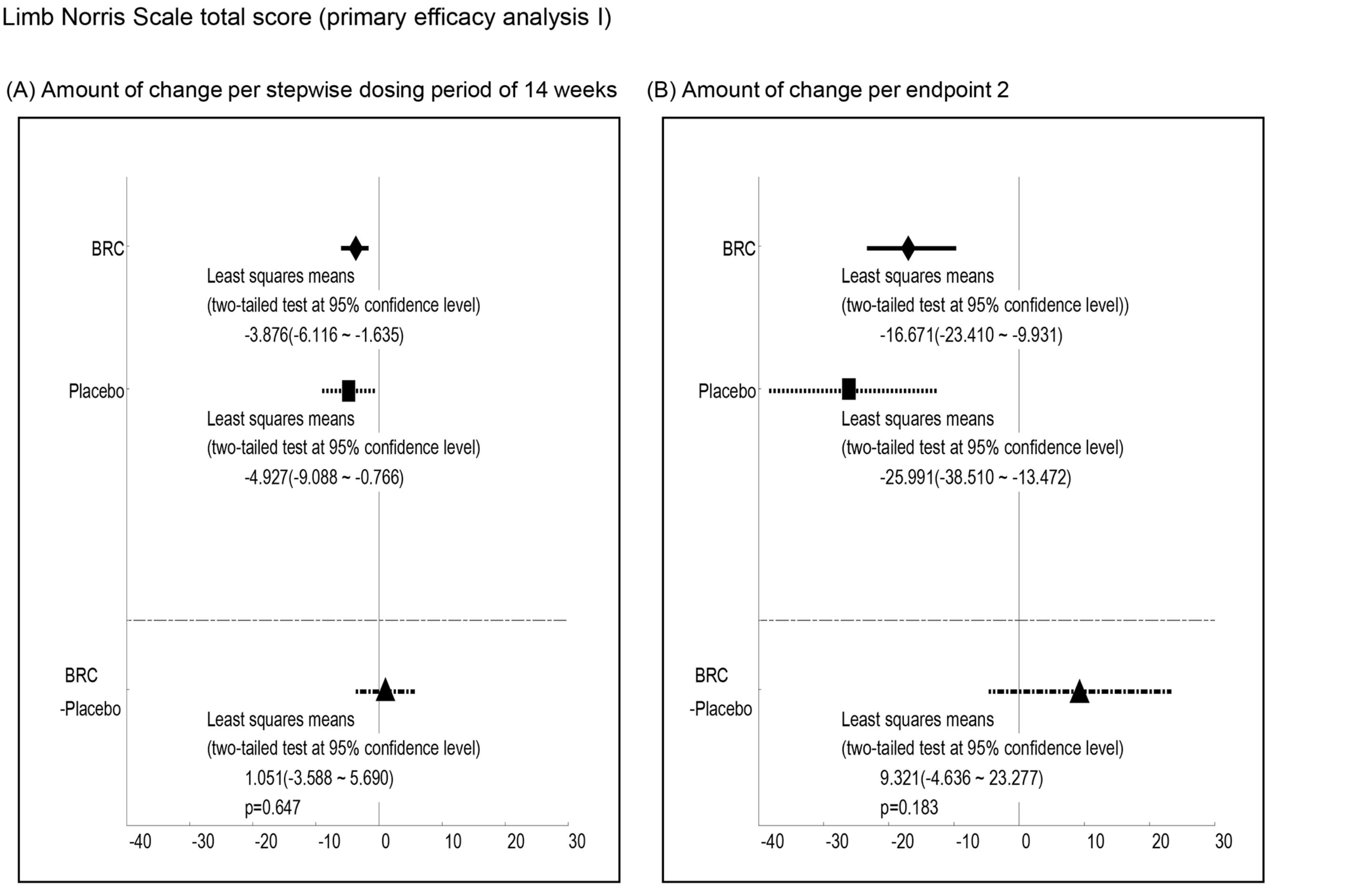

Fig 7. The results of primary efficacy analysis I on Grip strength (right side). In 1st endpoint analysis, the group of BRC treatment was significantly recovered compared to the group of placebo treatment ($p < 0.2$). Moreover, in 2nd endpoint analysis, the group of BRC tended to be recovered compared to the group of placebo.

doi:10.1371/journal.pone.0149509.g007

function and leads to increased survival and retention of motor function in *in vivo* models of ALS. Subsequently, EMPOWER, a phase 3 trial has shown that dexpramipexole although generally well tolerated did not differ from placebo in any prespecified efficacy endpoint measurement [22,23]. BRC is a unique member of the class of dopamine D2 receptor agonists with *in vitro* antioxidant properties [24–26]. Our previous studies have also shown that BRC upregulates NAIP and protects neuronal cells against oxidative insults independent of D2 receptor function as outlined below as well as delayings disease progression of ALS mouse. Interestingly, dopamine receptor antagonists (SCH23390: D1 dopamine receptor antagonist, Sulpiride: D2&D3 dopamine receptor antagonist, Raclopride: D2 dopamine receptor antagonist) did not affect BRC dependent anti-oxidative stress activity [27]. Interestingly, the alleviation of motor neuronal dysfunction in ALS mice by a post-onset administration of BRC was unchangeable by the presence or absence of Riluzole [27]. Furthermore, the results of this study imply that BRC in the presence of Riluzole is safe and well tolerated. Although this study had a comparative low number of ALS patients for an effectiveness evaluation, our results showed the BRC group showed trend toward efficacy suggesting it could be an effective

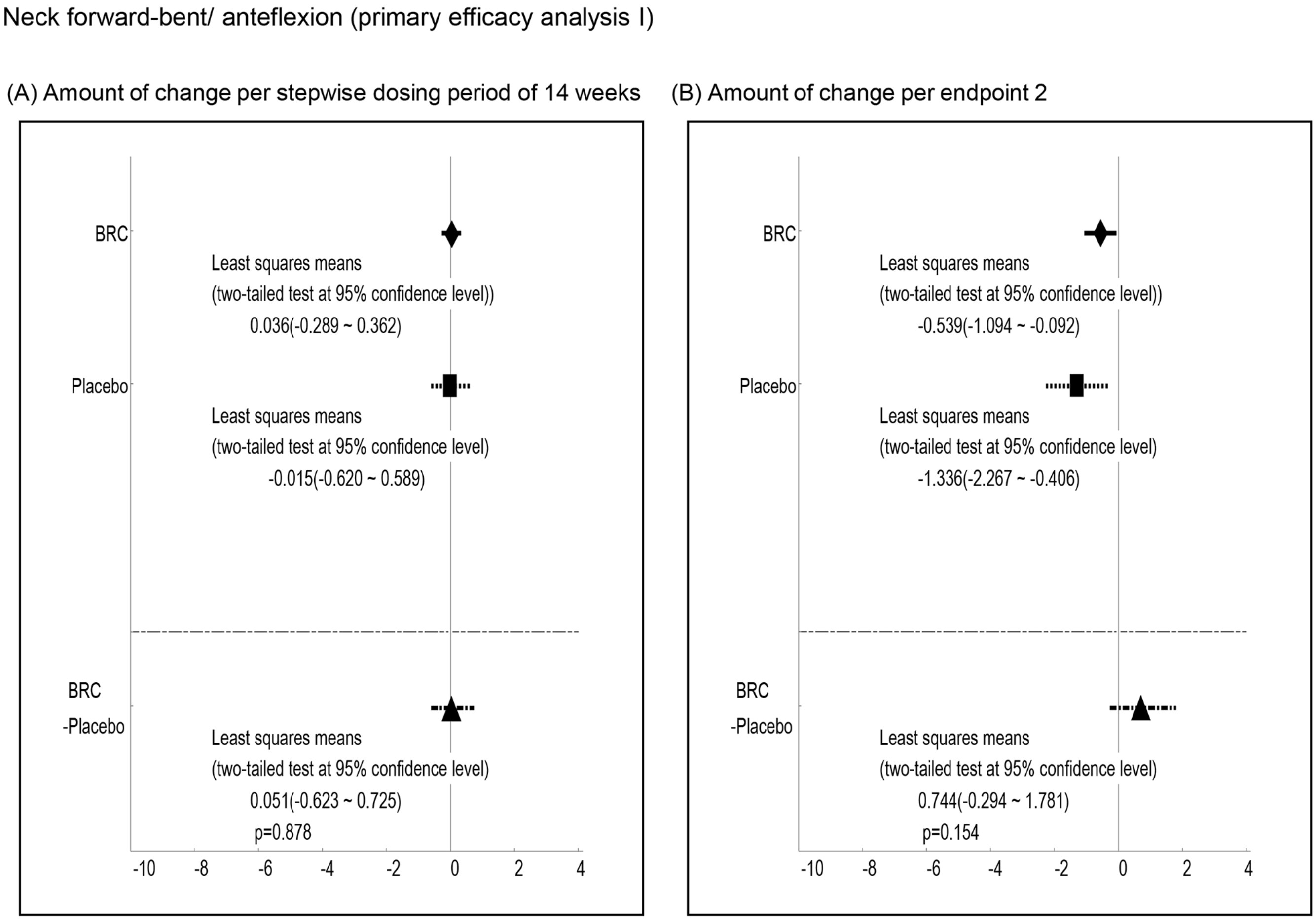

Fig 8. The results of primary efficacy analysis I on neck forward-bent/ anteflexion. In 1st endpoint analysis, the group of BRC treatment tended to be recovered compared to the group of placebo treatment. Moreover, in 2nd endpoint analysis, the group of BRC was significantly recovered compared to the group of placebo (p < 0.2).

doi:10.1371/journal.pone.0149509.g008

treatment for ALS. In both primary efficacy analyses I and II, the patients treated with BRC had greater residual functions of the upper and lower limbs as reflected in the ALSAQ40-communication, ALSAQ40-eating & drinking, and Grip strength scores when compared to placebos (Figs 4 and 5). Moreover, total ALSFRS-R scores and neck flexion test also tended to stability (Fig 6). The patients with early stage ALS showed the greatest effect on clinical scores. ALS is a complex neuromuscular degenerative disease, with an as yet undelineated and likely therapeutically refractory molecular pathology; the expectation of a panacea must be viewed as low. In this regard BRC protects cells against oxidative stress, which may underlie neuronal dysfunction (and/or degeneration) and manifestation of ALS but might not indeed is unlikely to account for all aspects of pathogenesis. It is conceivable for example that BRC sustains motoneuronal function at least in part through suppression of oxidative stress. BRC sustained both ALSFRS-R and grip strength until the 1st endpoint but not the 2nd. On the other hand, BRC treated cases revealed some preservation of the Limb Norris and degree of neck flexion with 15mg/day dosing at the 2nd endpoint. In addition placebo treated cases showed progressive deterioration of lower limb muscle function which BRC slowed (Fig 10). In addition to these

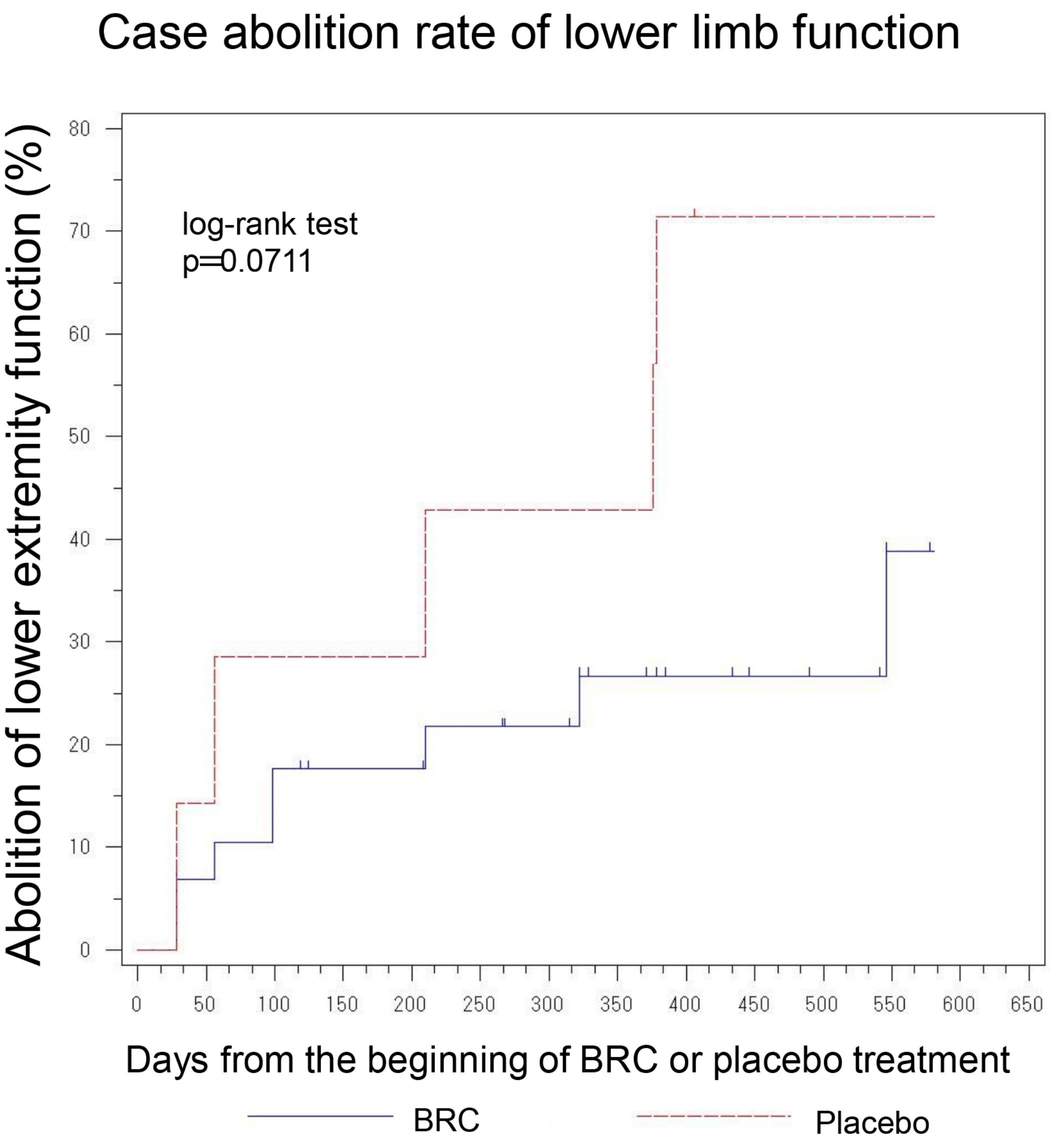

Fig 9. Alterations of differential white blood counts in ALS patients with or without BRC. Placebo treated ALS patients revealed significant increase of eosinophil. On the other hand, BRC treated ALS patients showed no increase of it (A). However, the number of the other blood cells stayed constant during the disease progression in both placebo and BRC treated group (B, C, D, E). LOCF: last observation carried forward. *P<0.05 BRC group vs Placebo group.

doi:10.1371/journal.pone.0149509.g009

encouraging functional effects, the progressive eosinophilia in the placebo but not BRC group was noteworth (Fig 9). This is first report of the cumulative increase of eosinophil but not other blood cells including basophil, neutrophil, and lymphocyte with ALS disease progression. Although the role of eosinophils in ALS is not clear, an upregulation of eosinophil-derived neurotoxin (END) from ALS patients but not Alzheimer or Parkinson diseases patients has been reported [28]. This may be attributable to neuronal inflammation via induction of oxidative stress. Whether eosinophil can be used as a biomarker for ALS, will emerge from closer analysis of the relationship between the eosinophil including END and ALS progression. In conclusion, the efficacy and safety of BRC should be further explored by further investigation of phase 2b/3 clinical trial.

Table 2. Summary of safety assessment.

Adverse events	BRC (n = 31)	Placebo (n = 7)
Infection and infestations	19 (61.3%)	3 (42.9%)
(nasopharyngitis)	(11 (35.5%))	(1 (14.3%))
Gastrointestinal disorders	14 (45.2%)	4 (57.1%)
(constipation)	(5 (16.1%))	(2 (28.6%))
(nausea)	(5 (16.1%))	(2 (28.6%))
Skin and subcutaneous tissue disorders	12 (38.7%)	1 (14.3%)
(eczema)	(4 (12.9%))	(0 (0%))
Adverse drug events		
Gastrointestinal disorders	6 (19.4%)	3 (42.9%)
(nausea)	3 (9.7%)	2 (28.6%)
Serious adverse events		
Respiratory, thoracic and mediastinal	3 (9.7%)	0 (0%)
(aspiration pneumonias)	(2 (6.5%))	(0 (0%))
Serious adverse drug events		
Gastrointestinal disorder (ischemic colitis)	1 (3.2%)	0 (0%)

doi:10.1371/journal.pone.0149509.t002

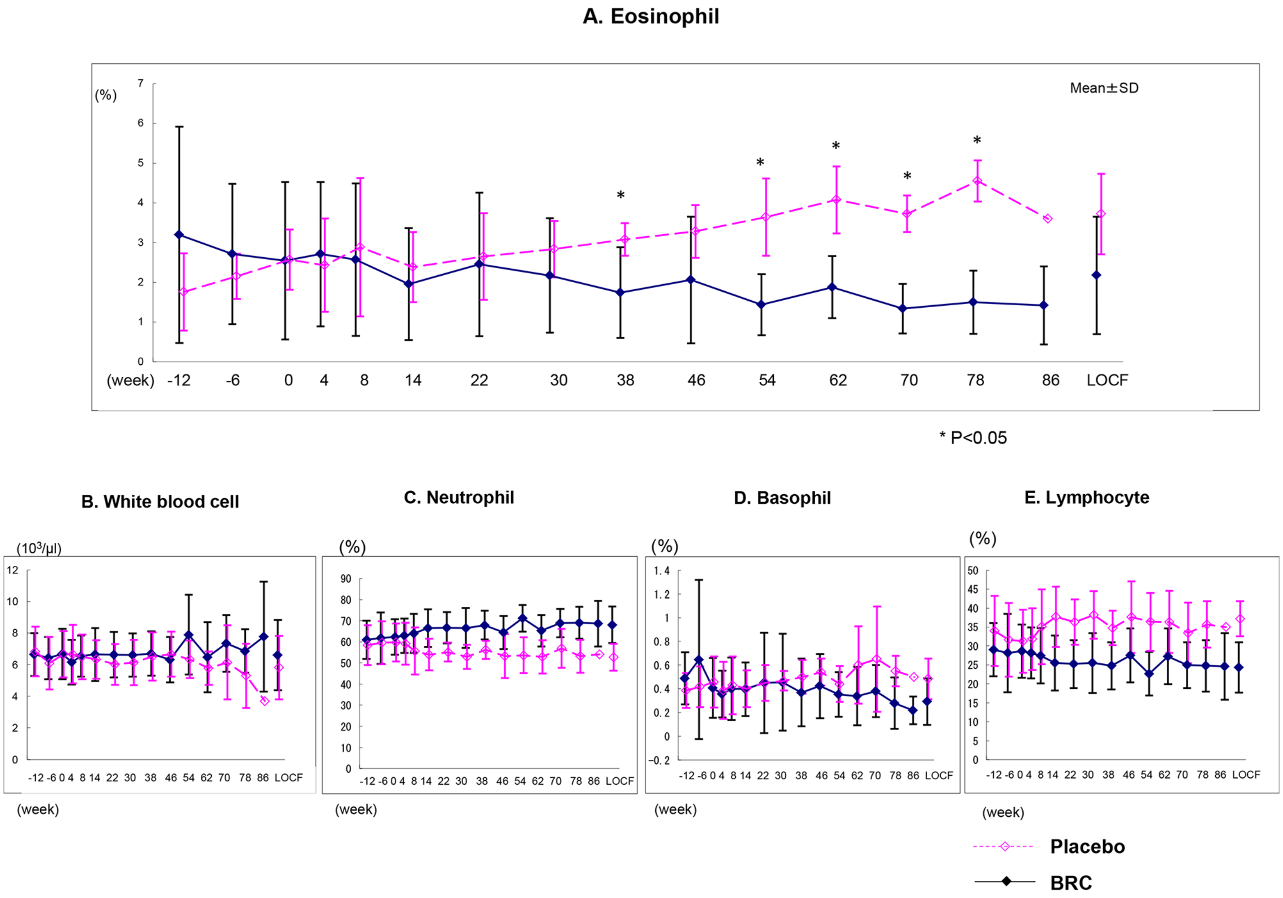

Fig 10. Case abolition rate of lower limb function. BRC signifiantly suppressed abolition rate of lower limb function in ALS compared to placebo.

doi:10.1371/journal.pone.0149509.g010

Supporting Information

S1 CONSORT Checklist.
(DOC)

S1 Protocol. IRB protocol approved for the trial.
(PDF)

Acknowledgments

We thank Prof. Alex MacKenzie (Children's Hospital of Eastern Ontario, Ottawa, Ontario, Canada) for fruitful discussion and encouragement in the preparation of this manuscript. This work was supported by Grant-of- Aid from Japanese Ministry of Health, Labor and welfare.

ALS consortium investigators

1. Study director: Joh-E Ikeda, Molecular Neurology-CMIC, Faculty of Medicine, Kitasato University, Department of Molecular Life Science, Faculty of Medicine, Tokai University, Kanagawa, Japan, ARC/Children's Hospital of Eastern Ontario, Department of Pediatrics, Faculty of Medicine, University of Ottawa, Ottawa, Ontario, Canada
2. Researchers: Shigeharu Takagi, Department of Neurology, Tokai University School of Medicine, Isehara, Japan; Fumihito Yoshii, Department of Neurology, Tokai University School of Medicine, Isehara, Japan; Shunya Takizawa, Department of Neurology, Tokai University School of Medicine, Isehara, Japan; Eiichiro Nagata, Department of Neurology, Tokai University School of Medicine, Isehara, Japan; Tomohide Ohnuki, Department of Neurology, Tokai University School of Medicine, Isehara, Japan; Yasuhisa Kitagawa, Department of Neurology, Tokai University School of Medicine, Isehara, Japan; Yasuo Iwasaki, Department of Neurology, Toho University Omori Medical Center, Tokyo, Japan; Yasunosuke Iwamoto, Department of Neurology, Toho University Omori Medical Center, Tokyo, Japan; Mieko Ogino, Department of Neurology, Kitasato University School of Medicine, Sagamihara, Japan
3. Study contributors: Wako Takahashi, Department of Neurology, Tokai University School of Medicine, Isehara, Japan; Yuko Ohnuki, Department of Neurology, Tokai University School of Medicine, Isehara, Japan; Kyuichiro Onoue, General Institute of Medicine, Tokai University, Isehara, Japan
4. ALS treatment plan evaluation committee: *Chairman*: Ichiro Kanazawa, Chief Medical officer, Grand Steward's Secretariat, Imperial Household Agency, Professor, International University of Health and Welfare Graduate School, Tochigi, Japan. *Members*: Shigeki Kuzuhara, Director, National Center of Neurology and Psychiatry, Tokyo, Japan; Yasuo Itoyama, Professor, Department of Neurology, Tohoku University Graduate School of Medicine, Sendai, Japan; Hajime Sobue, Professor, Department of Neurology, Nagoya University Graduate School of Medicine, Nagoya, Japan

Author Contributions

Conceived and designed the experiments: JEI. Performed the experiments: EN MO KI YK YI FY. Analyzed the data: EN FY JEI. Contributed reagents/materials/analysis tools: EN MO KI YK YI FY JEI. Wrote the paper: EN FY JEI.

References

1. Rowland LP, Shneider NA. Amyotrophic lateral sclerosis. *N Engl J Med*. 2001; 344: 1688–1700. PMID: 11386269
2. Sreedharan J, Brown RH Jr. Amyotrophic lateral sclerosis: problems and prospects. *Ann Neurol*. 2013; 74: 309–316. doi: 10.1002/ana.24012 PMID: 24038380
3. Barber SC, Mead RJ, Shaw PJ. Oxidative stress in ALS: a mechanism of neurodegeneration and a therapeutic target. *Biochem Biophys Acta*. 2006; 1762: 1051–1067. PMID: 16713195
4. Boillee S, Vande Velde C, Cleveland DW. ALS: a disease of motor neurons and their nonneuronal neighbors. *Neuron*. 2006; 52: 39–59. PMID: 17015226
5. Pasinelli P, Brown RH. Molecular biology of amyotrophic lateral sclerosis from genetics. *Nat Rev Neurosci*. 2006; 7: 710–723. PMID: 16924260
6. Barber SC, Shaw PJ. Oxidative stress in ALS: key role in motor neuron injury and therapeutic target. *Free Radic Biol Med*. 2010; 48: 629–641., 2010. doi: 10.1016/j.freeradbiomed.2009.11.018 PMID: 19969067
7. Rao SD, Yin HZ, Weiss JH. Disruption of glial glutamate transport by reactive oxygen species produced in motor neurons. *J Neurosci*. 2003; 23: 2627–2633. PMID: 12684448
8. Liu Y, Hao W, Dawson A, Liu S, Fassbender K. Expression of amyotrophic lateral sclerosis-linked SOD1 mutant increases the neurotoxic potential of microglia via TLR2. *J Biol Chem*. 2009; 284: 3691–3699. doi: 10.1074/jbc.M804446200 PMID: 19091752
9. Okada Y, Sakai H, Kohiki E, Suga E, Yanagisawa Y, Tanaka K, et al. A dopamine D4 recptor antagonist attenuates ischemia-induced neuronal cell damage via upregulaton of neuronal of neuronal apoptosis inhibitory protein. *J Cereb Blood Flow*. 2005; 25: 794–806.
10. Roy N, Mahadevan MS, Mclean M, Shutler G, Yaraghi Z, Farahani R, et al. The gene for apoptosis inhibitory protein is partially deleted in individuals with spinal muscle atrophy. *Cell*. 1995; 80: 167–178. PMID: 7813013
11. Liston P, Roy N, Tamai K, Lefebvre C, Baird S, Cherton-Horvat G, et al. Suppression of apoptosis in mammalian cells by NAIP and a related family of IAP genes. *Nature*. 1996; 379: 349–353. PMID: 8552191
12. Tanaka K, Okada Y, Kanno T, Otomo A, Yanagisawa Y, Shouguchi-Miyata J, et al. A dopamine receptor antagonist L-745870 suppresses microglia activation in spinal cord and mitigates the progression in ALS model mice. *Exp Neurol*. 2008; 211: 378–386. doi: 10.1016/j.expneurol.2008.02.004 PMID: 18423451
13. Tanaka K, Kanno T, Yanagisawa Y, Yasutake K, Inoue S, Hirayama N, et al. A novel acylaminoimidazole derivative, WN1316, alleviates disease progression via suppression of glial inflammation in ALS mouse model. *PLOS ONE*. 2014; 9: e87728. doi: 10.1371/journal.pone.0087728 PMID: 24498180
14. Lim JH, Kim KM, Kim SW, Hwang O, Choi HJ. Bromocriptine activates NQo1 via Nrf2-PI3K/Akt signaling: novel cytoprotective mechanism against oxidative damage. *Pharmacol Res*. 2008; 57: 325–331. doi: 10.1016/j.phrs.2008.03.004 PMID: 18455424
15. Ludolph AC, Bendotti C, Blaugrund E, Chio A, Greensmith L, Loeffler JP, et al. Guidline for preclinical animal research in ALS/MND: a consensus meeting. *Amyotroph Lateral Scler*. 2010; 11: 38–45. doi: 10.3109/17482960903545334 PMID: 20184514
16. Bensimon G, Lacomblez L, Meininger V. A controlled trial of riluzole in amyotrophic lateral sclerosis. ALS/Riluzole study group. *N Engl J Med*. 1994; 330: 585–591. PMID: 8302340
17. Miller RG, Mitchell JD, Lyon M, Moore DH. Riluzole for amyotrophic latral sclerosis (ALS)/motor neuron disease (MND). Cochrane Database Syst Rev. CD001447, 2007.
18. Mitsumoto H, Brookd BR, Silani V. Clinical trials in amyotrophic lateral sclerosis: why so many negative trials and how can trials be improved? *Lancet Neurol*. 2014; 13: 1127–1138. doi: 10.1016/S1474-4422(14)70129-2 PMID: 25316019
19. Brooks BR, Miller RG, Swash M, Munsat TL. El Escorial revised: revised criteria for the diagnosis of amyotrophic lateral sclerosis. *Amyotroph Lateral Scler Other Motor Neuron Disord*. 2000; 11: 497–501.
20. Benatar M. Lost in translation: treatment trials in the SOD1 mouse and in human ALS. *Neurobiol Dis*. 2007; 26: 1–13. PMID: 17300945
21. Turner BJ, Talbot K. Transgenics, toxicity and therapeutics in rodent models of disease progression in inherited amyotrophic lateral sclerosis. *Nat Neurosci*. 2008; 11: 251–253.
22. Cudkowicz M, Bozik ME, Ingersoll EW, Miller R, Mitsumoto H, Shefner J, et al. The effects of dexpramipexole (KNS-760704) in individuals with amyotrophic lateral sclerosis. *Lancet Neurol*. 2011; 17: 1652–1657.

23. Cudkowicz ME, van den Berg LH, Shefner JM, Mitsumoto H, Mora JS, Ludolph A, et al. Dexpramipexole versus placebo for patients with amyotrophic lateral sclerosis (EMPOWER): a randomized, double-blind, phase 3 trial. *Lancet Neurol*. 2013; 12: 1059–1067. doi: 10.1016/S1474-4422(13)70221-7 PMID: 24067398

24. Kondo T, Ito T, Sugita Y. Bromocriptine scavenges methamphetamine-induced hydroxyl radicals and attenuates dopamine depletion in mouse striatum. *Ann N Y Acad Sci*. 1994; 738: 222–229. PMID: 7832431

25. Ogawa N, Tanaka K, Asanuma M, Kawai M, Masumizu T, Kohno M, et al. Bromocriptine protects mice against 6-hydroxydopamine and scavenges hydroxyl free radicals in vitro. *Brain Res*. 1994; 657: 207–213. PMID: 7820619

26. Yoshikawa T, Minamiyama Y, Naito Y, Kondo M. Antioxidant properties of bromocriptine, a dopamine agonist. *J Neurochem*. 1994; 62: 1034–1038. PMID: 8113789

27. Tanaka K, Kanno T, Yanagisawa Y, Yasutake K, Hadano S, Yoshii F, et al. Bromocriptine methylate suppresses glial inflammation and moderates disease progression in a mouse model of amyotrophic lateral sclerosis. *Exp Neurol*. 2011; 232: 21–52.

28. Liu GT, Hwang CS, Hsieh CH, Lu CH, Chang SLY, Huang CF, et al. Eosinophil-derived neurotoxin is elevated in patients with amyotrophic lateral sclerosis. *Mediators Inflamm*. 2013; 2013: 1–7.
